# Interleukin-4 prevents increased endothelial permeability by inducing pericyte survival and modulating microglial responses in diabetic retinopathy

**DOI:** 10.3389/fendo.2025.1609796

**Published:** 2025-07-02

**Authors:** Jang-Hyuk Yun

**Affiliations:** College of Veterinary Medicine and Institute of Veterinary Science, Kangwon National University, Chuncheon, Gangwon, Republic of Korea

**Keywords:** pericytes, endothelial permeability, diabetic retinopathy, interleukin-4, signal transducer and activator of transcription 6, microglia functional states

## Abstract

**Introduction:**

Retinal vascular leakage due to increased endothelial permeability is a major contributor to the pathogenesis of diabetic retinopathy (DR) and visual impairment. Pericyte loss and microglia-mediated inflammation exacerbate this vascular dysfunction. Interleukin-4 (IL-4) is known for its anti-inflammatory and tissue-protective properties, but its role in DR remains unclear.

**Methods:**

We evaluated IL-4 expression and signaling in the retinas of streptozotocin-induced diabetic mice. *In vitro* assays were conducted under high-glucose and TNF-α conditions using retinal endothelial cells, pericytes, and microglia to assess IL-4’s effects on barrier function, cell viability, and inflammatory state. Pathway-specific analyses were performed to investigate PI3K/AKT and STAT6 signaling.

**Results:**

IL-4 expression and downstream signaling were significantly reduced in diabetic retinas. IL-4 promoted pericyte survival via PI3K/AKT activation and modulated microglial functional profiles through STAT6 signaling, favoring an anti-inflammatory phenotype. These effects contributed to restored endothelial barrier integrity and tight junction protein expression under diabetic stress conditions *in vitro*.

**Conclusion:**

IL-4 supports retinal vascular stabilization in DR by preserving pericyte viability and modulating microglial activity. These findings highlight IL-4 as a potential therapeutic target for preventing or slowing DR progression and warrant further preclinical investigation.

## 1 Introduction

Diabetic retinopathy (DR), a leading cause of vision impairment in humans (1), is the most prevalent microvascular complication in patients with diabetes (2). Vascular leakage in the retina, which disrupts the blood–retinal barrier and exacerbates retinal dysfunction, is a key contributor to vision impairment in DR (3). Pericytes are essential for maintaining blood vessel integrity through their interactions with endothelial cells (4). They upregulate tight junction proteins in endothelial cells, thereby reducing endothelial permeability and preventing vascular leakage (5, 6). Loss of pericytes is one of the early pathological changes in DR and is strongly associated with vascular leakage (7). Recent studies have shown that apoptosis of pericytes increases endothelial permeability in co-culture models with endothelial cells (8, 9). These findings indicate that promoting pericyte survival in DR could preserve retinal vascular integrity and mitigate vascular leakage in DR.

Interleukin (IL)-4, first identified in the mid-1980s, is a multifunctional cytokine primarily produced by activated T cells as well as by mast cells, basophils, and eosinophils (10). The molecular weight of IL-4 varies between 12 to 20 kDa due to variable natural glycosylation (11), and it shares sequence homology, cell surface receptors, intracellular signaling pathways, and functional properties with IL-13 (12, 13). IL-4 is essential for defining the Th2 lymphocyte phenotype and regulating cell apoptosis, proliferation, and gene expression in diverse cell types, including lymphocytes, macrophages, fibroblasts, and epithelial cells (13–17). IL-4 is also associated with the regulation of microglial functional states by promoting transcriptional and phenotypic programs associated with tissue remodeling and neuroprotection (18, 19). IL-4 enhances neuronal survival and mitigates neuroinflammatory responses, underscoring its broader role in maintaining central nervous system (CNS) homeostasis (20, 21). Previous study, including our own, have confirmed that pro-inflammatory cytokines, such as IL-6 and IL-1β are elevated in diabetic retinas and contribute to vision impairment in DR (8, 22–24). However, the role of IL-4 in regulating pericyte survival, endothelial permeability, or its overall involvement in DR remains unclear.

Microglia, as central regulators of neuroinflammatory responses, are highly responsive to changes in their environment (25). In DR, microglia often adopt pro-inflammatory phenotypes characterized by the production of cytokines such as tumor necrosis factor-alpha (TNF-α) and IL-1β (26–28). These cytokines exacerbate neuronal damage and vascular instability by promoting pericyte apoptosis and increasing endothelial permeability (8, 27). IL-4 has been shown to induce anti-inflammatory and tissue-supportive microglial phenotypes in other disease models (29). Based on these findings, we hypothesized that IL-4 may counteract the pro-inflammatory retinal microenvironment in DR by modulating microglial states and promoting pericyte survival.

To test this hypothesis, in this study, we aimed to determine whether IL-4 can protect pericytes from apoptosis and restore endothelial barrier function under diabetic conditions by employing a combination of *in vivo* (streptozotocin-induced diabetic mouse model) and *in vitro* co-culture systems involving human retinal microglia, pericytes, and endothelial cells. Our findings revealed decreased IL-4 levels in the retina of diabetic mice. Mechanistically, IL-4 induced pericyte survival directly through the phosphoinositide 3-kinase/protein kinase (PI3K/Akt) pathway and indirectly by modulating microglial phenotypes toward a homeostatic, anti-inflammatory state. Furthermore, IL-4 prevented diabetes-induced increases in endothelial permeability by inhibiting pericyte apoptosis and influencing microglial responses. These findings suggest the potential of IL-4 in preserving vascular integrity in the diabetic retina.

## 2 Materials and methods

### 2.1 Cell cultures

Human pericytes from the placenta (PromoCell, Heidelberg, Germany), human primary retinal microvascular endothelial cells (HRMECs; ACBRI, Kirkland, WA, USA), and human brain astrocytes (ACBRI) were maintained in M199 medium (HyClone, Logan, UT, USA) containing 20% fetal bovine serum (FBS), Dulbecco’s modified eagle medium (DMEM; Thermo Fisher Scientific, Waltham, MA, USA) containing 10% FBS, and pericyte medium containing growth factors (PromoCell), respectively. HMO6, an immortalized human microglial cell line, was obtained as described in a previous study (22) and maintained in DMEM containing 10% FBS. The cells were incubated at 37°C in a 5% CO_2_ atmosphere.

### 2.2 Reagents and antibodies

Recombinant human IL-4, TNF-α, and mouse IL-4 enzyme-linked immunosorbent assay (ELISA) kits were purchased from R&D Systems (Minneapolis, MN, USA). Human IL-1β, IL-6, IL-23, TNF-α, arginase-1 (Arg-1), IL-10, and insulin-like growth factor-1 (IGF-1) ELISA kits were purchased from Thermo Fisher Scientific. 3-(4,5-Dimethylthiazol-2-yl)-2,5-diphenyltetrazolium bromide (MTT), AS1517499 (a selective signal transducer and activator of transcription 6 [STAT6] inhibitor), wortmannin (a PI3K inhibitor that suppresses Akt pathway activation), PD98059 (a MEK1 inhibitor that blocks the extracellular signal-regulated kinase 1/2 [Erk1/2] pathway), streptozotocin (STZ), Evans blue dye, bovine serum albumin (BSA), glucose, and mannitol were purchased from Millipore-Sigma (St. Louis, MO, USA). Other reagents and antibodies used were: anti-STAT6, anti-STAT5, anti-Akt, anti- Erk1/2, anti-cleaved caspase-3, anti-Bcl-2-associated X protein (Bax), anti-B-cell lymphoma 2, anti-Bcl-extra-large (Bcl-xL), anti-phosphorylated STAT6 (anti-phospho-STAT6), anti-phospho-STAT5, anti-phospho-Akt, and anti-phospho-Erk1/2 (Cell signaling Technology [CST], Danvers, MA, USA); anti-β-tubulin and peroxidase-conjugated secondary antibodies (Santa Cruz Biotechnology, Dallas, TX, USA); anti-zonula occludens-1 (ZO-1) and anti-occludin (Thermo Fisher Scientific).

### 2.3 Cell viability assay

Cell viability was measured using the MTT assay kit (Millipore-Sigma). Briefly, 5 × 10^3^ cells/well were plated in 96-well plates for 48 h and treated with IL-4 (50 ng/mL) or TNF-α (100 mg/mL) for 48 h under normal glucose (5 mmol/L glucose), high mannitol (20 mmol/L mannitol and 5 mmol/L glucose), and high glucose (25 mmol/L glucose) conditions. After treatment, 10 μL of MTT solution (5 mg/mL) was added to each well containing 100 μL of culture medium, resulting in a final MTT concentration of 0.45 mg/mL. The cells were incubated for an additional 3 h at 37°C. Subsequently, the supernatant was carefully removed, and the resulting formazan crystals were solubilized in 100 μL of dimethyl sulfoxide (DMSO). Absorbance was measured at 570 nm using a microplate reader.

### 2.4 Apoptosis assay

The apoptotic effect was assessed using the Annexin-V-fluorescein isothiocyanate (FITC)/propidium iodide (PI) double-staining assay, following protocols supplied with Muse AnnexinV & Dead Cell Assay kit (Millipore-Sigma). Approximately 3 × 10^5^ cells were treated with IL-4 or TNF-α for 48 h under normal glucose, high mannitol, and high glucose conditions. After incubation, cells were collected in 1 mL of medium containing 1% FBS. Subsequently, the cell suspension (100 μL) was mixed with FITC AnnexinV/PI and vortexed gently for 5 s. The mixture was then incubated for 20 min at room temperature and analyzed using the Muse cell analyzer (Millipore-Sigma). Flow cytometry data were visualized as dot plots, with untreated cells serving as the control. All experiments were performed in triplicate.

### 2.5 Western blot analysis

Cells were collected and lysed in radioimmunoprecipitation assay (RIPA) buffer supplemented with a protease inhibitor cocktail. Equal amounts of protein (30 μg per lane) were loaded onto 4–20% gradient SDS-polyacrylamide gels for electrophoresis and subsequently transferred to nitrocellulose membranes. The membranes were blocked for 1 h at room temperature in Tris-buffered saline containing 0.1% Tween-20 (TBST) and 5% non-fat dry milk, and then incubated overnight at 4°C with primary antibodies in the same blocking buffer (TBST with 5% non-fat dry milk). The following primary antibodies were used: anti-phospho-STAT6 (1:1000, CST; #9361), anti-STAT6 (1:1000, CST; #5397), anti-phospho-Akt (1:1000, CST; #4060), anti-Akt (1:1000, CST; #4691), anti-phospho-Erk1/2 (1:1000, CST; #9101), anti-Erk1/2 (1:1000, CST; #9102), anti-cleaved caspase-3 (1:1000, CST; #9664), anti-Bax (1:1000, CST; #2772), anti-Bcl-2 (1:1000, CST; #2870), anti-Bcl-xL (1:1000, CST; #2764), anti-ZO-1 (1:500, Thermo Fisher Scientific; #61-7300), and anti-Occludin (1:500, Thermo Fisher Scientific; #71-1500). β-tubulin (1:2000, Santa Cruz Biotechnology; #sc-5274) was used as a loading control.

After washing three times with TBST (10 min each), the membranes were incubated for 1 h at room temperature with horseradish peroxidase (HRP)-conjugated secondary antibodies (1:2000, Santa Cruz Biotechnology; #sc-2005 or #sc-2004) diluted in TBST containing 5% non-fat dry milk.

Protein bands were visualized using an HRP-based enhanced chemiluminescence substrate (Thermo Fisher Scientific) and exposed to film. Densitometric analysis of the bands was performed using ImageJ software (National Institutes of Health, Bethesda, MD, USA).

### 2.6 Quantitative reverse transcription polymerase chain reaction

Total RNA was extracted from cultured cells and tissues using the RNeasy Plus Mini kit (Qiagen, Hilden, Germany), following the manufacturer’s protocol. Briefly, 1 μg of total RNA was reverse-transcribed into cDNA using murine leukemia virus reverse transcriptase in the presence of 2.5 μM oligo-dT primers and 1 mM dNTPs. qRT-PCR was carried out using SYBR Green master mix (Applied Biosystems, CA, USA) on an Applied Biosystems StepOnePlus Real-Time PCR system. Each reaction was carried out in a final volume of 20 μL, containing 2 μL of synthesized cDNA, 10 μL of 2× SYBR Green master mix, and 0.4 μM of each primer (forward and reverse). The thermal cycling conditions were as follows: initial denaturation at 95°C for 10 min, followed by 40 cycles of 95°C for 15 s and 60°C for 60 s. A melting curve analysis was performed after amplification to confirm the specificity of the PCR products. The primer sequences used in this study are listed in **Table 1**. Each sample was run in triplicate, and relative gene expression levels were calculated using the 2^–ΔΔCt^ method. Expression values were normalized to *Actb* (mouse) or *ACTB* (human) as internal reference genes.

**Table 1:** Primer sequences used for qPCR analysis.

Gene	Species	Forward Primer (5'–3')	Reverse Primer (5'–3')
Il4	Mouse	ATGGGTCTCAACCCCCAGCTAGT	GCTCTTTAGGCTTTCCAGGAAGTC
Ilb	Mouse	CCCATTAGACAACTGCACTAC	GATTCTTTCCTTTGAGGCCC
Il6	Mouse	CTTCTTGGGACTGATGCTGGT	GGTCTGTTGGGAGTGGTATCC
Il10	Mouse	CGGGAAGACAATAACTG	CATTTCCGATAAGGCTTGG
Il12b	Mouse	ATCGTTTTGCTGGTGTCTCC	CATCTTCTTCAGGCGTGTCA
Il13	Mouse	CCTCTGACCCTTAAGGAGCTT	ATGTTGGTCAGGGAATCCAG
Il18	Mouse	ACAACTTTGGCCGACTTCAC	GGGTTCACTGGCACTTTGAT
Actb	Mouse	CCAGGCATTGCTGACAGGAT	AGCCACCGATCCACACAGAG
ILB	Human	ACGCTCCGGGACTCACAGCA	TGAGGCCCAAGGCCACAGGT
IL6	Human	TGACAAACAAATTCGGTACATCCT	AGTGCCTCTTTGCTGCTTTCAC
IL23A	Human	GTGGGACACATGGATCTAAGAGAAG	TTTGCAAGCAGAACTGACTGTTG
TNFA	Human	CACAGTGAAGTGCTGGCAAC	AGGAAGGCCTAAGGTCCACT
ARG1	Human	TGGACAGACTAGGAATTGGCA	CCAGTCCGTCAACATCAAAACT
IL10	Human	GACTTTAAGGGTTACCTGGGTTG	TCACATGCGCCTTGATGTCTG
IGF1	Human	TGTGGAGACAGGGGCTTTTA	CCTGCACTCCCTCTACTTGC
ACTB	Human	GGGAAATCGTGCGTGACATT	AGTTTCGTGGATGCCACAGG

### 2.7 Permeability assay

The Transwell inserts (Corning, NY, USA) were inverted, and then pericytes (5 × 10^4^ cells/cm^2^) or HMO6 (5 × 10^4^ cells/cm^2^) were seeded on the bottom side of the inserts for 2 h to form an *in vitro* blood-retina barrier model. After the pericytes or HMO6 were attached, the inserts were inverted, and HRMECs (1 × 10^5^ cells/cm^2^) were seeded on the top side, followed by incubation for 24 h. Both cells were exposed to normal or high glucose conditions, with or without IL-4, TNF-α, wortmannin, or AS1517499 for 48 h. To assess endothelial permeability, 0.5% Evans blue dye was added to the upper chamber and incubated for 1 h at 37°C. The optical density of the medium collected from the bottom chamber was measured spectrophotometrically at 650 nm using a microplate reader (Tecan, Mannedorf, Switzerland).

### 2.8 Animals

All animal experiments were conducted in accordance with the National Institutes of Health Guide for the Care and Use of Laboratory Animals and were approved by the Institutional Animal Care and Use Committee of Kangwon National University.

Eight-week-old male C57BL/6 mice were obtained from Central Laboratory Animal Inc. (Seoul, South Korea). Only male mice were used to avoid variability caused by the estrous cycle, which can influence inflammatory responses and vascular permeability. This approach ensured consistency in evaluating the effects of IL-4 in DR. Diabetes was induced by fasting the mice for 6 hours, followed by a single intraperitoneal injection of STZ (180 mg/kg) dissolved in 10 mmol/L citrate buffer (pH 4.5). Age-matched control mice received citrate buffer alone. Blood glucose levels were measured 72 h after injection via tail vein sampling using a glucometer. Mice with glucose levels >300 mg/dL were considered diabetic and included in subsequent experiments. Although multiple low-dose STZ injections are commonly used to model autoimmune Type 1 diabetes, in this study, a single high-dose protocol was selected to achieve rapid and consistent hyperglycemia, which is widely used in mechanistic studies of DR. Animals were monitored daily and sacrificed by CO&_2_ inhalation at 3 months post-STZ injection to evaluate retinal pathophysiology during the chronic phase of hyperglycemia. Immediately after euthanasia, eyes were enucleated, and retinas were carefully isolated on ice for subsequent qRT-PCR, ELISA, and western blotting.

### 2.9 Microglia-conditioned media preparation

HMO6 microglial cells were treated with IL-4 (50 ng/mL) alone or in combination with AS1517499 (1 μM), wortmannin (1 μM), or PD98059 (25 μM) for 48 h. After treatment, culture supernatants were collected and centrifuged at 3,000 rpm for 10 min at 4°C to remove detached cells and cellular debris. The resulting supernatants were carefully transferred to fresh tubes and used as microglia-conditioned media for subsequent experiments involving pericytes or endothelial cells. No additional ultracentrifugation or extracellular vesicle isolation was performed.

### 2.10 Statistical analyses

Statistical analyses were performed using GraphPad Prism (version 9; GraphPad Software, San Diego, CA, USA). For comparisons involving more than two groups or multiple factors, one-way or two-way analysis of variance (ANOVA) followed by Tukey’s *post-hoc* tests was applied. An unpaired two-tailed Student’s t-test assuming unequal variances was used for comparisons between two groups. A P-value of < 0.05 was considered statistically significant. Quantitative data are presented as mean ± standard deviation (SD).

## 3 Results

### 3.1 Levels of IL-4 expression and the activity of its downstream signaling pathway decreased in diabetic retinas

**Figure 1:**
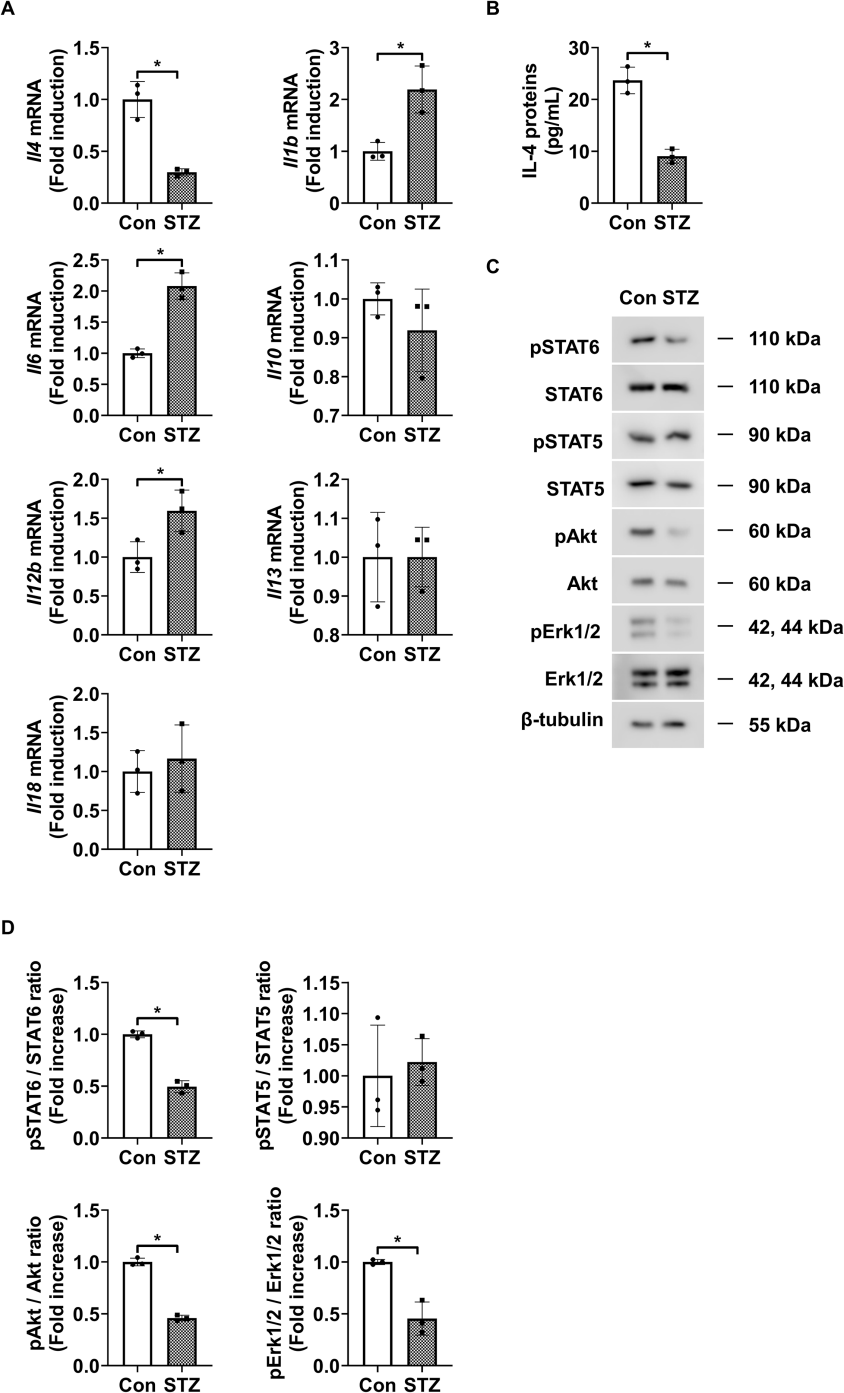
IL-4 expression and downstream signaling activity in retinas of STZ-induced diabetic mice. **(A)** mRNA levels of *Il4*, *Il1b*, *Il6*, *Il10*, *Il12b*, *Il13*, and *Il18* measured using qRT-PCR in retinas from 3-month-old STZ-induced diabetic mice (STZ) and age-matched control mice (Con). **(B)** IL-4 protein levels measured using ELISA in retinal lysates from 3-month-old STZ and Con mice. **(C)** Expression of phosphorylated and total STAT6, STAT5, Akt, and Erk1/2 in retinal lysates assessed using western blot analysis. β-tubulin was used as a loading control. **(D)** Densitometric quantification of the immunoblot bands in **(C)**. Bar graphs in A–C show mean ± SD (n = 3). *P < 0.05 assessed using Student’s *t*-test. IL-4, interleukin-4; STZ, streptozotocin; qRT-PCR, quantitative reverse transcription polymerase chain reaction; Con, control; ELISA, enzyme-linked immunosorbent assay; pSTAT6, phosphorylated signal transducer and activator of transcription 6; pSTAT5, phosphorylated STAT5; pAkt, phosphorylated protein kinase B; pErk1/2, phosphorylated extracellular signal-regulated kinase 1/2; β-tubulin, beta-tubulin.

We first investigated the expression profiles of various interleukins in the retinas of STZ-induced diabetic mice. The level of *Il4* mRNA was significantly reduced in the retinas of diabetic mice compared to those in those of control mice, whereas the mRNA levels of *Il1b*, *Il6*, and *IL12b* were significantly elevated (**Figure 1A**). In contrast, *Il10, Il13*, and *Il18* expression did not show significant differences between diabetic and control retinas. Next, we performed ELISA and western blot analyses to assess IL-4 expression and the activation status of its downstream signaling molecules, such as STAT6, STAT5, Akt, and Erk1/2. Expression of IL-4 was significantly reduced (**Figure 1B**), along with a significant reduction in the phosphorylation of STAT6, Akt, and Erk1/2 in STZ-induced diabetic retinas (**Figures 1C, D**). However, the expression of phospho-STAT5 did not show significant alteration.

### 3.2 IL-4 directly induces pericyte survival

**Figure 2:**
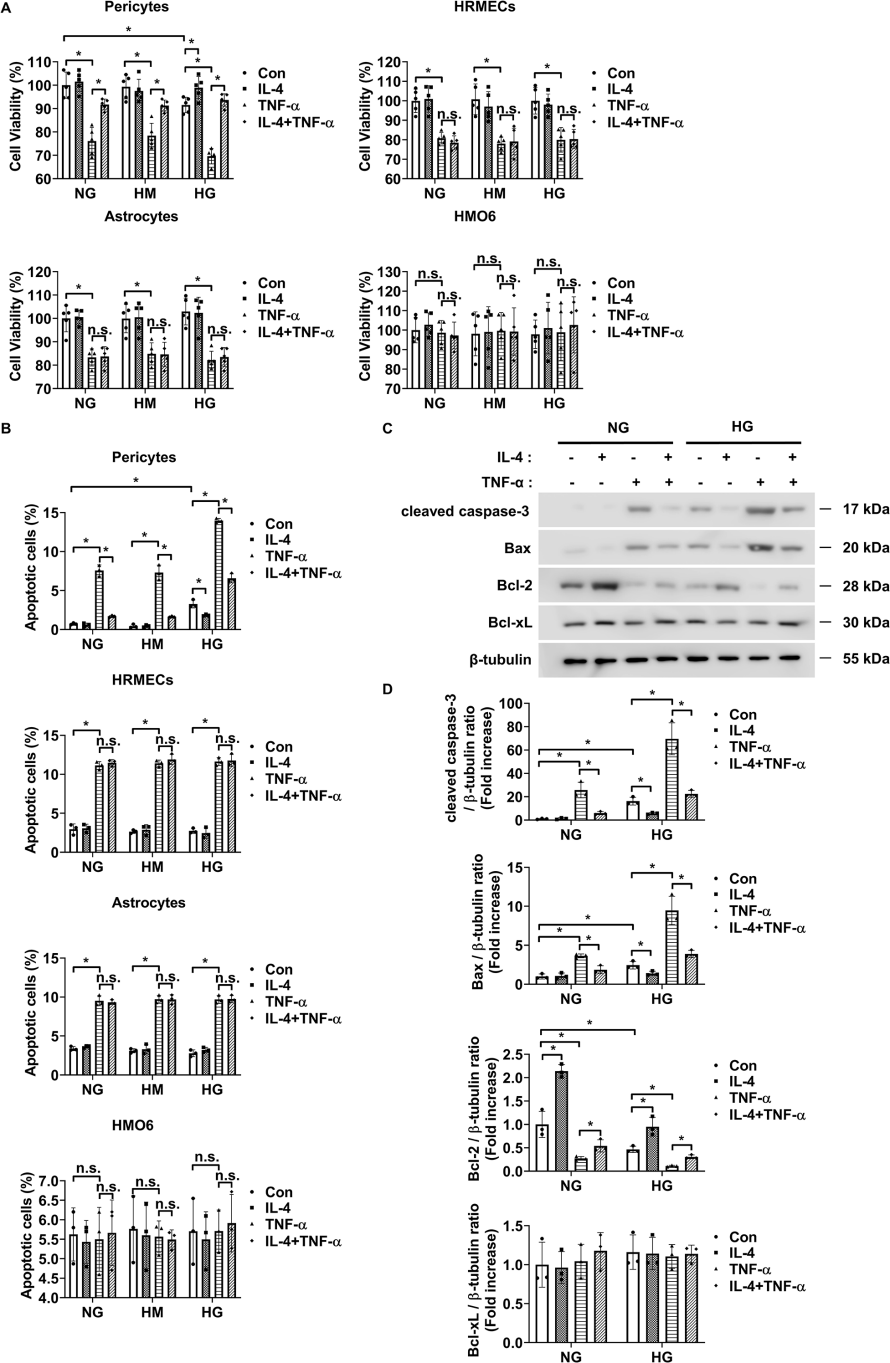
IL-4 promotes pericyte survival under high-glucose conditions. **(A, B)** Cell viability assessed using the MTT assay **(A)**, and apoptosis analyzed using annexin V/PI staining followed by flow cytometry **(B)** in pericytes, HRMECs, astrocytes, and HMO6 cells cultured under normal glucose (NG; 5 mmol/L glucose), high mannitol (HM; 20 mmol/L mannitol + 5 mmol/L glucose), or high glucose (HG; 25 mmol/L glucose) conditions with or without IL-4 (50 ng/mL) and TNF-α (100 ng/mL) treatments for 48 h **(C)** Expression levels of cleaved caspase-3, Bax, Bcl-2, and Bcl-xL in pericyte lysates assessed using western blot analysis under NG or HG conditions, with or without IL-4 and TNF-α treatment for 48 h β-tubulin was used as a loading control. **(D)** Quantitative densitometric analysis of western blot bands in **(C)**, showing protein expression normalized to β-tubulin. In **(A, B, D)** bar graphs represent mean ± SD (n = 3). Statistical analyses were performed using two-way ANOVA followed by Tukey’s *post hoc* test. *P < 0.05. IL-4, interleukin-4; HRMECs, human retinal microvascular endothelial cells; HMO6, human microglia clone 6; TNF-α, tumor necrosis factor-alpha; MTT, (3-(4,5-dimethylthiazol-2-yl)-2,5-diphenyltetrazolium bromide) assay; PI, propidium iodide; Bax, Bcl-2-associated X protein; Bcl-2, B-cell lymphoma 2; Bcl-xL, B-cell lymphoma-extra large; β-tubulin, beta-tubulin. n.s., not significant.

MTT and annexin-V/PI flow cytometric analyses revealed that IL-4 restored the high glucose- and TNF-α-induced reductions in cell viability of pericytes but did not affect the cell viability of HRMECs, astrocytes, and HMO6 (**Figure 2A**). Similarly, IL-4 reduced high glucose- and TNF-α-induced pericyte apoptosis but did not affect the apoptosis of HRMECs, astrocytes, and HMO6 (**Figure 2B**). Next, we performed western blot analysis to evaluate the effect of IL-4 on the pericyte apoptotic pathway. High glucose and TNF-α treatment increased the expression levels of cleaved caspase-3 and pro-apoptotic Bax proteins and decreased those of anti-apoptotic proteins, including Bcl-2 (**Figures 2C, D**). However, treatment with IL-4 reversed these high glucose- and TNF-α-induced changes, restoring the levels of cleaved caspase-3, Bax, and Bcl-2 in pericytes (**Figures 2C, D**).

### 3.3 IL-4 induced pericyte survival by activating Akt

**Figure 3:**
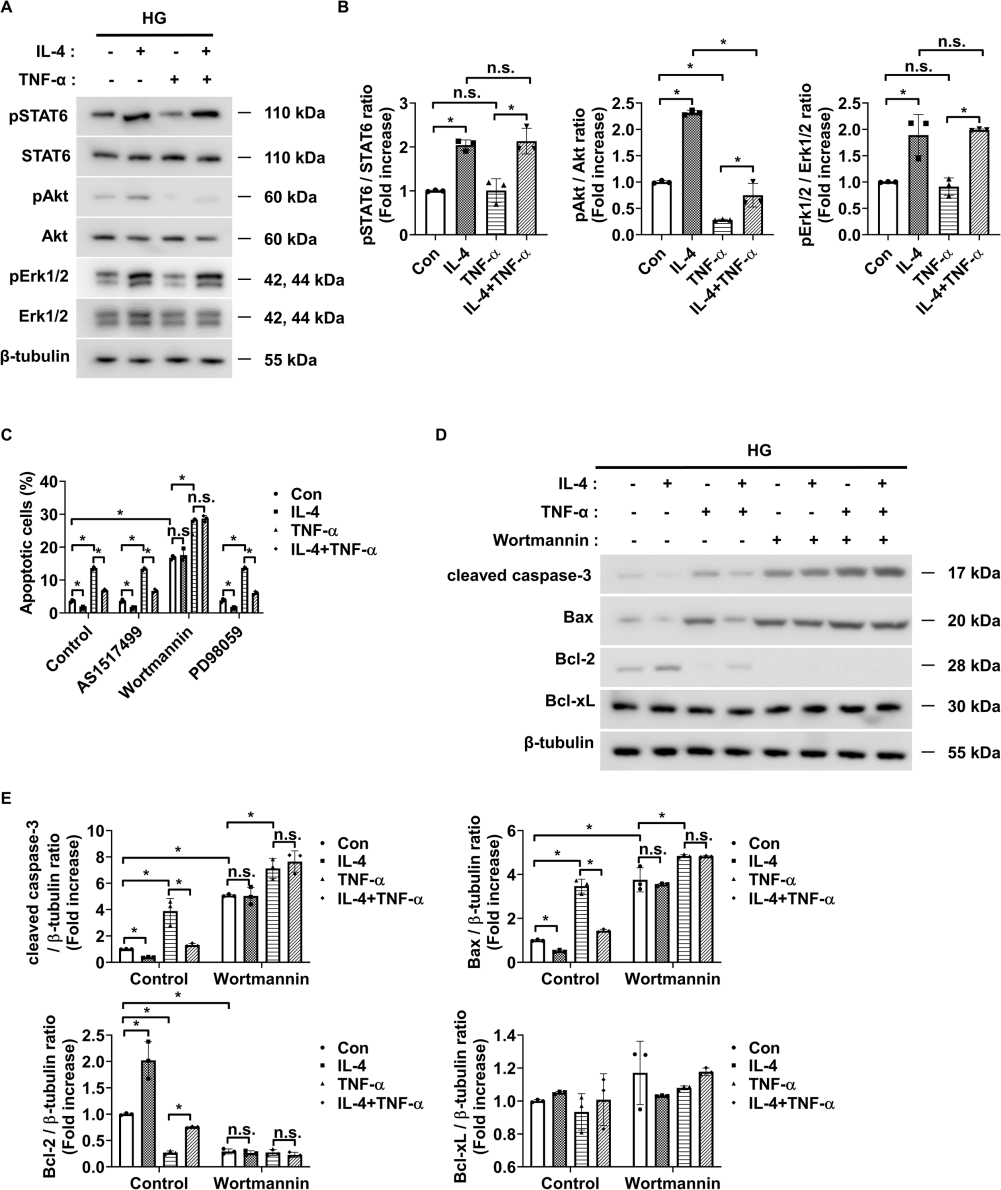
IL-4 promotes pericyte survival through the Akt signaling pathway. **(A)** Western blot analysis of phospho-STAT6 (pSTAT6), STAT6, phospho-Akt (pAkt), Akt, phospho-Erk1/2 (pErk1/2), and Erk1/2 in lysates from pericytes treated with or without IL-4 (50 ng/mL) and TNF-α (100 ng/mL) for 30 min under high glucose (HG; 25 mmol/L) conditions. β-tubulin was used as a loading control. **(B)** Densitometric quantification of protein expression in **(A)**, normalized to β-tubulin. **(C)** Cell apoptosis assessed using annexin-V/PI staining and flow cytometric analysis in pericytes pretreated with AS1517499 (1 μM), wortmannin (1 μM), or PD98059 (25 μM) for 1 h, followed by IL-4 and/or TNF-α treatment for 48 h under HG conditions. **(D)** Western blot analysis of cleaved caspase-3, Bax, Bcl-2, and Bcl-xL in lysates from pericytes pretreated with wortmannin (1 μM) for 1 h and subsequently treated with or without IL-4 and TNF-α for 48 h under high glucose conditions. β-tubulin was used as a loading control. **(E)** Densitometric quantification of protein expression in **(D)**, normalized to β-tubulin. In **(B, C, E)** bar graphs represent mean ± SD (n = 3). Statistical analysis was performed using two-way ANOVA followed by Tukey’s *post hoc* test. n.s., not significant; *P < 0.05. IL-4, interleukin-4; TNF-α, tumor necrosis factor-alpha; pSTAT6, phosphorylated STAT6; pAkt, phosphorylated Akt; pErk1/2, phosphorylated extracellular signal-regulated kinase 1/2; Bax, Bcl-2-associated X protein; Bcl-2, B-cell lymphoma 2; Bcl-xL, B-cell lymphoma-extra large; PI, propidium iodide; β-tubulin, beta-tubulin.

To investigate the signaling mechanism underlying IL-4-induced pericyte survival in diabetic retinas, we focused on pathways previously reported to be activated by IL-4 and associated with cell survival, including STAT6, Erk1/2, and Akt (30–32). Under high glucose conditions, IL-4 treatment significantly increased the phosphorylation of STAT6, Akt, and Erk1/2 in pericytes (**Figures 3A, B**). TNF-α treatment alone markedly reduced Akt phosphorylation without affecting that of STAT6 or Erk1/2 (**Figures 3A, B**). Co-treatment with IL-4 and TNF-α inhibited the IL-4-induced increase in phospho-Akt, while that of STAT6 and Erk1/2 remained unaffected (**Figures 3A, B**). To further evaluate the involvement of these pathways in IL-4-mediated pericyte survival, we used specific pharmacological inhibitors–AS1517499, wortmannin, and PD98059 completely prevented IL-4-induced STAT6 phosphorylation, IL-4-induced Akt phosphorylation, and IL-4-induced Erk1/2 phosphorylation, respectively, in pericytes (**Supplementary Figures 1**-**3**). Of these, wortmannin abolished IL-4-induced increase in pericyte survival (**Figure 3C**), decrease in cleaved caspase-3 and pro-apoptotic Bax levels, and increase in anti-apoptotic Bcl-2 levels in pericytes (**Figures 3D, E**).

### 3.4 IL-4 modulates microglial inflammatory responses via STAT6 signaling

**Figure 4:**
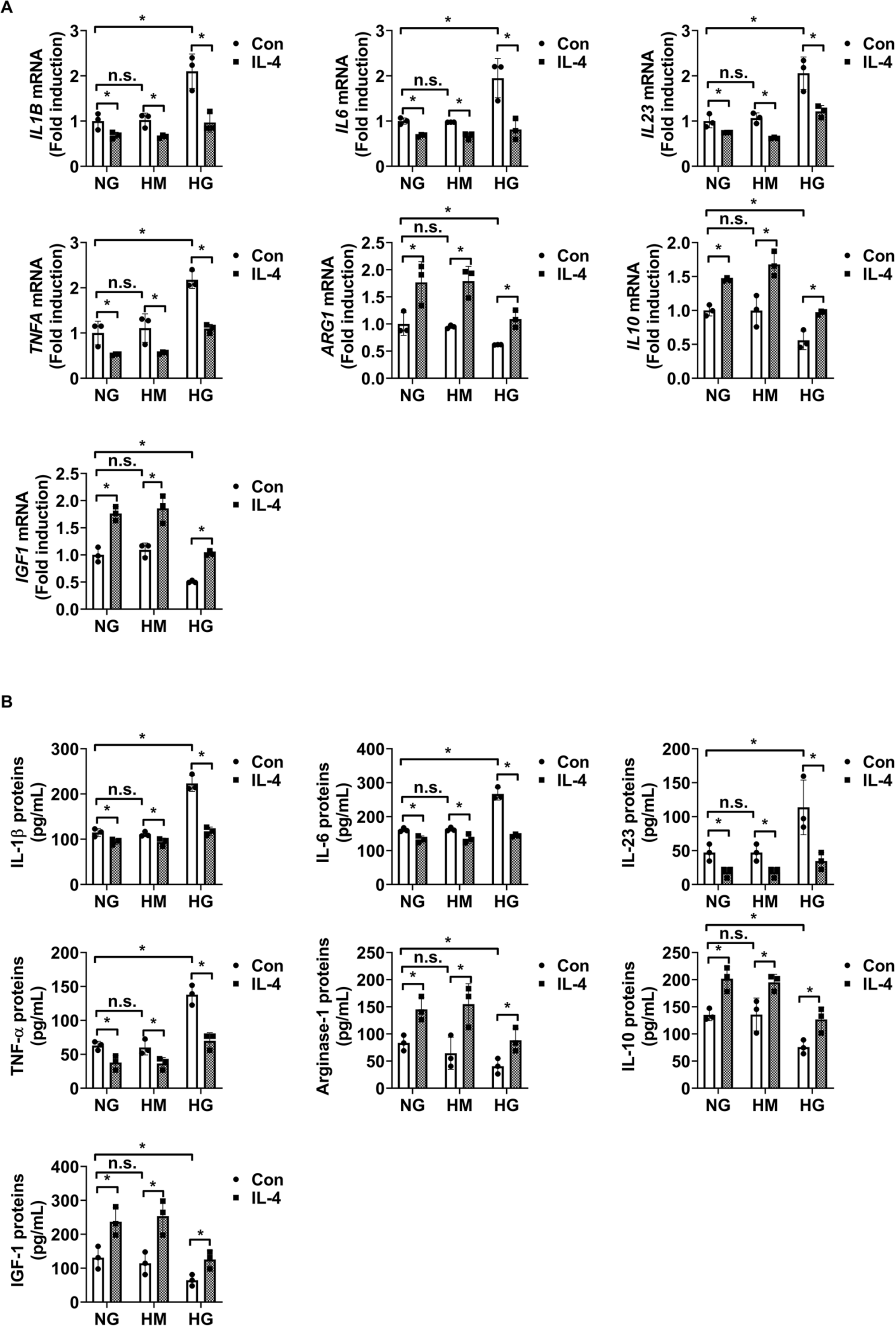
IL-4 regulates inflammatory factor expression in HMO6 microglia under high glucose conditions. **(A, B)** Expression levels of *IL1B, IL6, IL23, TNFA*, *Arg1*, *IL10*, and *IGF1* mRNA **(A, B)** proteins in HMO6 cells cultured under normal glucose (NG; 5 mmol/L glucose), high mannitol (HM; 20 mmol/L mannitol + 5 mmol/L glucose), or high glucose (HG; 25 mmol/L glucose) conditions with or without IL-4 (50 ng/mL) for 48 h measured using qRT-PCR **(A)** and ELISA **(B)**. Bar graphs show mean ± SD (n = 3). Statistical analyses were performed using two-way ANOVA followed by Tukey’s *post hoc* test. n.s., not significant; *P < 0.05. Arg-1, arginase-1; IL-10, interleukin-10; IGF-1, insulin-like growth factor 1.

Next, we evaluated the effects of IL-4 on microglial inflammatory responses under diabetic conditions by analyzing mRNA and protein levels of representative pro-inflammatory cytokines (IL-1β, IL-6, IL-23, and TNF-α) and anti-inflammatory or tissue-supportive factors (arginase-1 [Arg-1], IL-10, and IGF-1) in HMO6 microglial cells using qRT-PCR and ELISA. IL-4 treatment alone significantly decreased the expression of pro-inflammatory cytokines and increased the expression of Arg-1, IL-10, and IGF-1 under both normal glucose and high mannitol conditions, indicating an inherent immunomodulatory role independent of hyperglycemic stress (**Figures 4A, B**). Exposure to high glucose conditions induced a pro-inflammatory phenotype in HMO6 cells, characterized by elevated IL-1β, IL-6, IL-23, and TNF-α, and reduced levels of Arg-1, IL-10, and IGF-1. Co-treatment with IL-4 under high glucose conditions reversed these changes, restoring gene and protein expression levels toward those observed under normal glucose conditions (**Figures 4A, B**).

**Figure 5:**
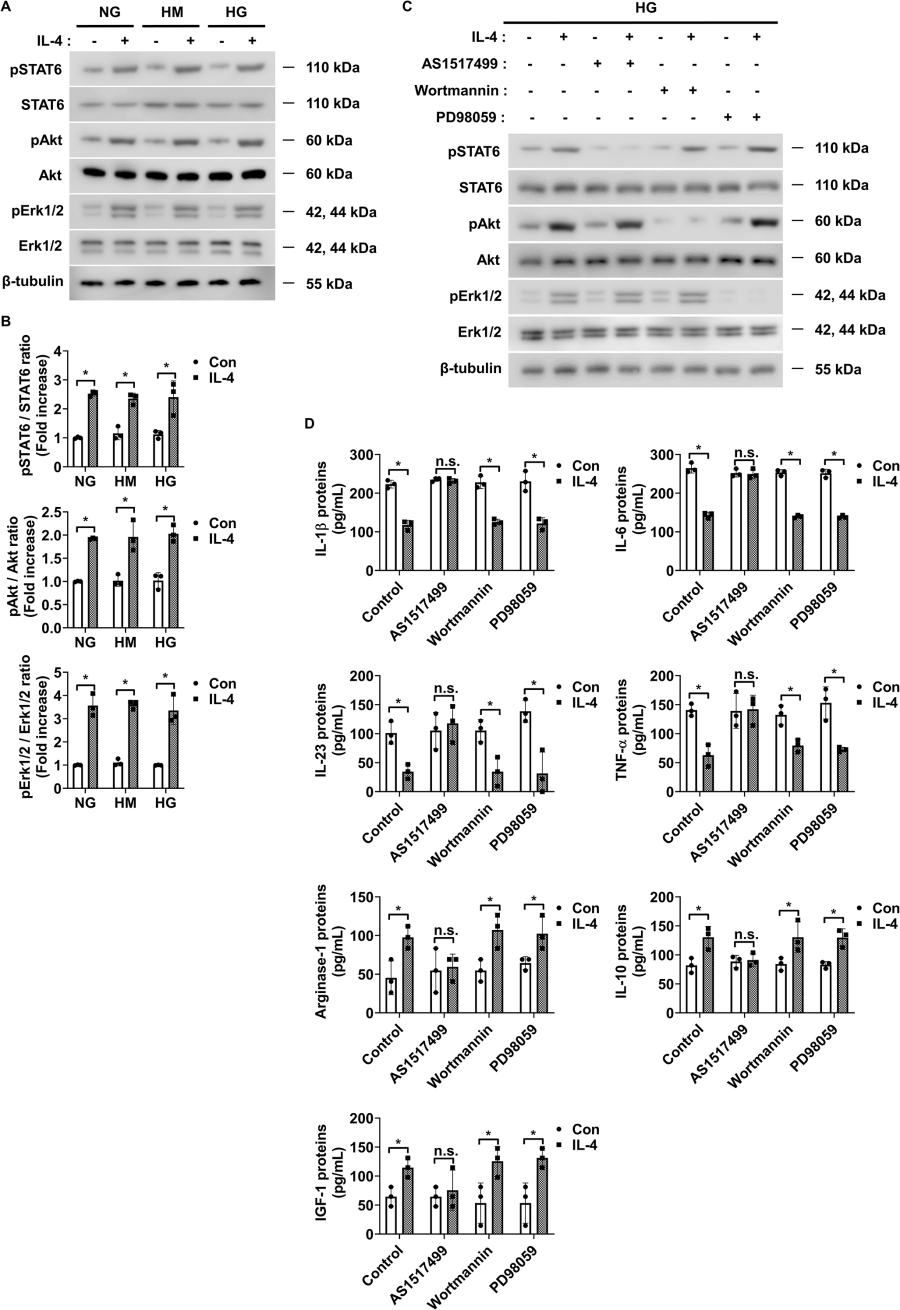
IL-4 activates STAT6 signaling in HMO6 microglia under high glucose conditions. **(A)** Western blot analysis of phosphorylated and total STAT6, Akt, and Erk1/2 performed on lysates from HMO6 cells treated with or without IL-4 (50 ng/mL) for 30 min under NG, HM, or HG conditions. β-tubulin was used as a loading control. **(B)** Densitometric quantification of protein bands in **(A)** normalized to β-tubulin. **(C)** Western blot analysis of pSTAT6, STAT6, pAkt, Akt, pErk1/2, and Erk1/2. HMO6 cells were pretreated with AS1517499 (1 μM), wortmannin (1 μM), or PD98059 (25 μM) for 1 h and then exposed to IL-4 for 30 min under HG conditions. β-tubulin was used as a loading control. **(D)** Protein levels of IL-1β, IL-6, IL-23, TNF-α, Arg-1, IL-10, and IGF-1 in culture supernatants measured using ELISA. HMO6 cells were pretreated as in **(C)** and exposed to IL-4 for 48 h under HG conditions. In B and D, bar graphs show mean ± SD (n = 3). Statistical analyses were performed using two-way ANOVA followed by Tukey’s *post hoc* test. n.s., not significant; *P < 0.05.

To identify the intracellular pathways responsible for IL-4-mediated immunoregulation, we assessed the expression of phosphorylated and total STAT6, Akt, and Erk1/2 using western blot analysis. IL-4 increased STAT6, Akt, and Erk1/2 phosphorylation in HMO6 cells under both normal and high glucose conditions, indicating that IL-4-driven activation of these signaling pathways is independent of extracellular glucose concentration (**Figures 5A, B**). In contrast, high glucose alone does not alter phosphorylation levels of these proteins, suggesting that the glucose-induced pro-inflammatory shift does not act through direct modulation of STAT6, Akt, or Erk1/2 activity. Instead, IL-4 appears to act as an external regulator capable of overriding glucose-induced changes in microglial function by selectively activating these signaling cascades. To further delineate the signaling pathway mediating immunomodulatory effects of IL-4, we used pharmacological inhibitors targeting each pathway: AS1517499 (STAT6 inhibitor), wortmannin (PI3K/Akt inhibitor), and PD98059 (Erk1/2 inhibitor). Each inhibitor selectively blocked IL-4-induced phosphorylation of its corresponding target (**Figure 5C**; **Supplementary Figure 4A**). Only AS1517499 abolished the IL-4-mediated suppression of pro-inflammatory cytokines and enhancement of Arg-1, IL-10, and IGF-1 expression under high glucose conditions (**Figure 5D**; **Supplementary Figure 5A**).

### 3.5 IL-4 is indirectly involved in pericyte survival through HMO6 cells in DR

IL-4 reduced the secretion of TNF-α and IL-1β, known to induce pericyte apoptosis from HMO6 microglial cells under high glucose conditions (**Figures 4A, B**). Therefore, we evaluated whether IL-4 confers indirect protection to pericytes by modulating microglial signaling.

**Figure 6:**
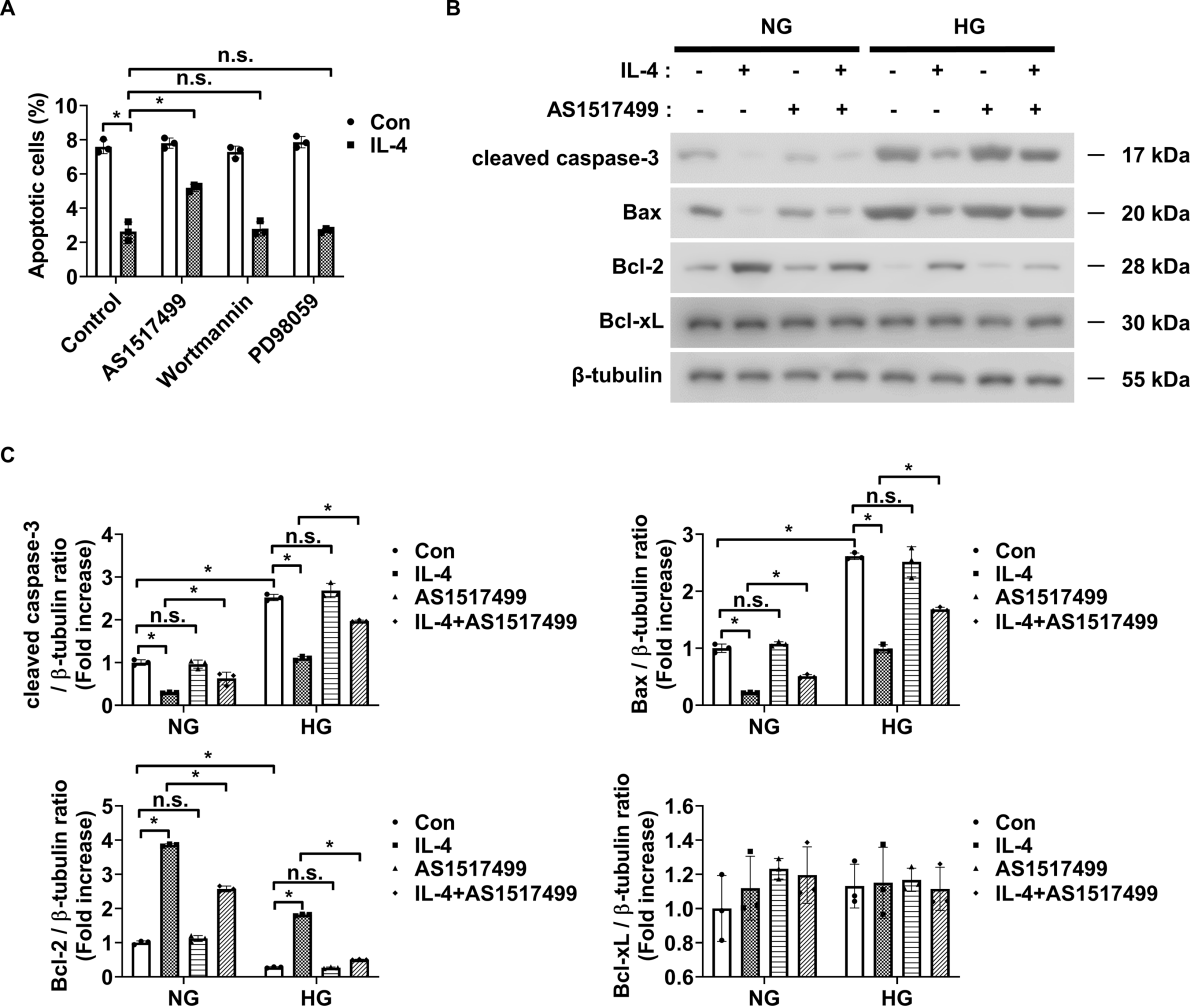
Conditioned media from IL-4-treated microglia affects pericyte apoptosis. **(A)** Apoptosis in HMO6 cells pretreated with AS1517499 (1 μM), wortmannin (1 μM), or PD98059 (25 μM) for 1 h and then cultured with or without IL-4 for 48 h under HG conditions assessed using annexin-V/PI staining and flow cytometry. **(B)** Expression of cleaved caspase-3, Bax, Bcl-2, and Bcl-xL in HMO6 cells pretreated with AS1517499 and exposed to IL-4 under NG or HG conditions for 48 h assessed using western blot analysis. Conditioned media were applied to pericytes for 48 h β-tubulin was used as a loading control. **(C)** Densitometric quantification of protein bands in **(B)** normalized to β-tubulin. In A and C, data show mean ± SD (n = 3). Statistical analyses were performed using two-way ANOVA followed by Tukey’s *post hoc* test. n.s., not significant; *P < 0.05.

To assess this, pericytes were cultured with conditioned media collected from HMO6 cells treated with IL-4, alone or in combination with pathway-specific inhibitors. Conditioned media from IL-4-treated HMO6 cells significantly reduced pericyte apoptosis under high glucose conditions (**Figure 6A**). This reduction was abolished when IL-4 was co-administered with AS1517499 in HMO6 cells (**Figure 6A**). In contrast, co-treatment with wortmannin or PD98059 did not affect the IL-4-induced protective effect (**Figure 6A**). Consistently, western blot analysis showed that conditioned media from IL-4-treated HMO6 cells decreased cleaved caspase-3 and pro-apoptotic Bax levels and increased Bcl-2 levels in pericytes (**Figures 6B, C**). These effects were reversed when IL-4 was combined with AS1517499 but not with wortmannin or PD98059.

### 3.6 IL-4 prevents endothelial permeability through pericyte survival and modulation of microglial inflammatory responses in DR

Next, we assessed changes in endothelial permeability under high glucose conditions using co-cultures of pericytes and HRMECs or HMO6 and HRMECs to evaluate the effect of IL-4 on endothelial barrier integrity. First, we investigated whether IL-4 directly influences endothelial permeability in DR. High glucose alone did not affect endothelial permeability or the expression of ZO-1 and occludin in HRMECs (**Supplementary Figures 6A–C**). However, TNF-α significantly increased endothelial permeability and reduced ZO-1 and occludin expression under both normal and high glucose conditions, suggesting a dominant, glucose-independent effect of TNF-α on barrier disruption (**Supplementary Figures 6A–C**). IL-4 treatment did not directly alter endothelial permeability or affect ZO-1 and occludin expression in HRMECs (**Supplementary Figures 6A–C**), indicating that IL-4 did not directly influence endothelial cell permeability in the diabetic retina model.

**Figure 7:**
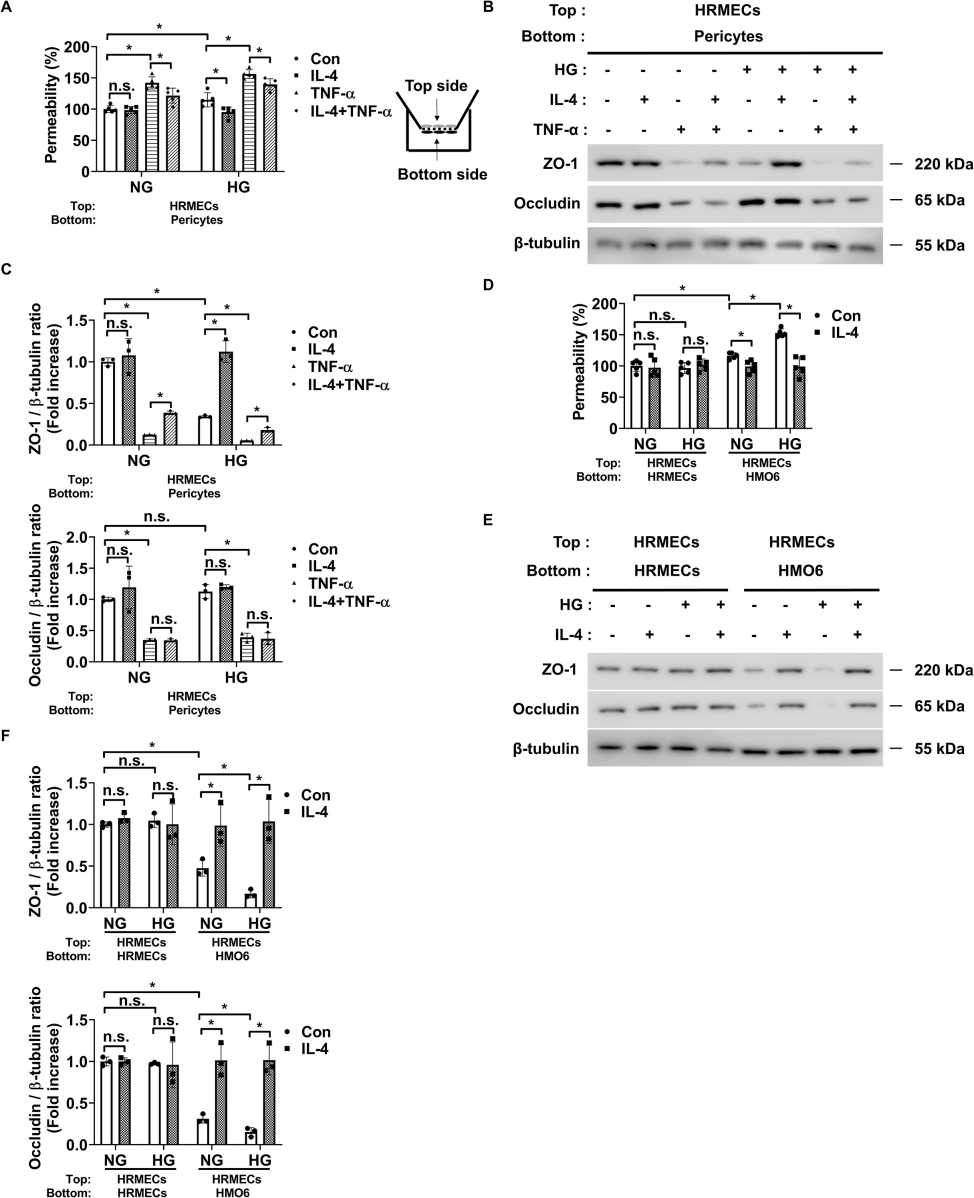
IL-4 affects endothelial permeability through actions on pericytes and microglia. **(A)** Permeability measured using Evans blue dye assay (n = 5). **(B)** Western blot analysis of ZO-1 and occludin in lysates from HRMECs. β-tubulin was used as a loading control. In A and B, pericytes and HRMECs were co-cultured on opposite sides of a Transwell insert and exposed to NG or HG conditions with or without IL-4 (50 ng/mL) and TNF-α (100 ng/mL) for 48 h. **(C)** Densitometric quantification of protein bands in **(B)** normalized to β-tubulin. **(D)** Permeability measured using Evans blue dye assay (n = 5). **(E)** Western blot analysis of ZO-1 and occludin in lysates from HRMECs. In **(D, E)** HRMECs were co-cultured with HRMECs or HMO6 cells on opposite sides of a Transwell insert and exposed to NG or HG conditions with or without IL-4 for 48 h β-tubulin was used as a loading control. **(F)** Densitometric quantification of protein bands in **(E)** normalized to β-tubulin. In A, C, D, and F, data represent mean ± SD (n = 3). Statistical analyses were performed using two-way ANOVA followed by Tukey’s *post hoc* test. n.s., not significant; *P < 0.05.

In our previous study, we confirmed that preventing pericyte apoptosis under DR conditions prevents the increase in endothelial permeability by preventing the decrease in ZO-1 expression but not occludin (5). Therefore, we speculated that IL-4 might prevent the increase in endothelial permeability by preventing the decrease in ZO-1 expression in DR when co-cultured with pericytes and HRMECs. Our findings showed that in pericyte-HRMEC co-cultures, IL-4 prevented the increase in endothelial permeability and decrease in ZO-1 expression in HRMECs under high glucose conditions and TNF-α exposure (**Figures 7A–C**). IL-4 also exerted a significant protective effect against TNF-α–induced endothelial permeability and ZO-1 reduction even under normal glucose conditions, although the effect was more pronounced under high glucose (**Figures 7A–C**). This finding suggests that the barrier-protective function of IL-4 is not strictly glucose-dependent but may be amplified in a diabetic-like environment. However, IL-4 did not affect occludin expression levels in these co-cultures (**Figures 7B, C**). Furthermore, the addition of wortmannin to the pericyte-HRMEC co-cultures restored the effect of IL-4 on endothelial permeability and ZO-1 expression in HRMECs (**Supplementary Figures 7A–C**).

Next, we explored whether the regulatory effects of IL-4 on microglial inflammatory states could influence endothelial permeability. Under high glucose, microglia upregulated TNF-α, which disrupted endothelial barrier function by downregulating ZO-1 and occludin (**Figures 4A, B**; **Supplementary Figures 6A–C**). Co-culture of HRMECs with IL-4-treated HMO6 cells under hyperglycemic conditions preserved both ZO-1 and occludin expression and suppressed the increase in endothelial permeability (**Figures 7D–F**). However, inhibition of the STAT6 pathway using AS1517499 abrogated the protective effects of IL-4, indicating that the immunoregulatory function of IL-4 is STAT6-dependent in microglia (**Supplementary Figures 8A–C**). Furthermore, IL-4 significantly reduced TNF-α expression in HMO6-HRMEC co-cultures under high glucose, while STAT6 inhibition reversed this effect (**Supplementary Figure 8D**).

## 4 Discussion

Pericyte apoptosis is one of the earliest pathological features of DR, observed in both patients with diabetes and diabetic animal models (9, 33). As pericytes are essential for maintaining vascular integrity, preventing their loss is critical to mitigating vision impairment in DR. However, the specific proteins and signaling mechanisms that protect pericytes from apoptosis in DR remain largely undefined. ILs are the key contributors to retinal pathology in diabetes—in our previous study, we identified IL-6 and IL-1β as important mediators in DR progression (8, 22). However, the role of IL-4, which is reduced in the diabetic retina, remains poorly understood. In this study, we focus on the relationship between reduced IL-4 levels and pericyte loss in DR. Our findings demonstrate that IL-4 directly enhances pericyte survival via activation of the Akt signaling pathway in DR.

Microglia, the resident immune cells of the CNS, including the retina, exhibit a ramified morphology under normal conditions (34–36). In response to pathological stimuli, they transition to an activated amoeboid form (37), with the nature and extent of microglial transformation varying by disease stage and microenvironmental cues (38). These context-dependent transformations contribute to the pathogenesis of various neurodegenerative and retinal diseases (39, 40). Under inflammatory conditions, microglia adopt a pro-inflammatory phenotype characterized by the release of cytokines such as IL-1β, IL-6, IL-23, and TNF-α (20, 41–43). Conversely, anti-inflammatory stimuli like IL-4, IL-10, IL-13, TGF-β, or glucocorticoids promote an immunoregulatory phenotype that supports tissue repair and homeostasis characterized by increased expression of Arg-1, IL-10, and IGF-1 (20, 42, 43). In DR, microglia predominantly adopt a pro-inflammatory state (28), releasing cytokines that contribute to pericyte loss and increased endothelial permeability (8, 27, 44).

Our findings showed significantly reduced levels of IL-4 in diabetic retinas (**Figures 1A, B**) and that IL-4 supplementation reversed the pro-inflammatory microglial phenotype while enhancing expression of tissue-supportive markers (**Figures 4A, B**), leading to improved pericyte survival and reduced endothelial permeability (**Figures 6A–C**; **Supplementary Figures 8A–D**). Furthermore, we also showed that IL-4 had no direct effect on endothelial barrier function in HRMEC monocultures, suggesting its protective role is context-dependent. However, in HRMEC-pericyte co-cultures, IL-4 attenuated TNF-α-induced barrier dysfunction, particularly under high-glucose conditions, indicating its indirect action via pericyte stabilization. Similarly, in HRMEC-microglia co-cultures, IL-4 reduced microglial TNF-α production through a STAT6-dependent mechanism, thereby protecting endothelial tight junction integrity. These findings highlight the importance of cell–cell interactions in mediating vascular protective effects of IL-4 in DR. A recent meta-analysis reported elevated IL-4 levels in the aqueous and vitreous humor of patients with proliferative DR (45), which could be attributed to a compensatory response to chronic inflammation in late stage disease. In contrast, our study suggests that IL-4 deficiency in early DR contributes to disease progression. Together, these findings underscore the complexity of IL-4 dynamics across DR stages and suggest that exogenous IL-4 supplementation may still offer therapeutic benefits by reinforcing anti-inflammatory signaling and promoting vascular stability.

Mechanistically, IL-4 exerts distinct effects in different cell types: in pericytes, it activates the PI3K/Akt pathway to enhance survival, while in microglia, it activates STAT6 to suppress pro-inflammatory signaling. Although IL-4 can activate Akt, Erk1/2, and STAT6 in various cell types (30–32), our data indicate that only STAT6 is required for IL-4-mediated regulation of microglia in DR (**Figure 5D**; **Supplementary Figure 5A**). This cell type-specificity likely reflects differences in receptor composition, the availability of signaling intermediates, or downstream transcriptional machinery between pericytes and microglia, consistent with the findings of previous studies on pleiotropic cytokines. Previous studies using the oxygen-induced retinopathy (OIR) model reported elevated *Il4* mRNA expression, increased STAT6 and PPAR-γ activation, and upregulation of tissue-repair-associated microglial genes (46). However, these studies did not confirm whether IL-4 directly activates STAT6 in microglia or whether STAT6 is necessary for the effects of IL-4. In the present study, pharmacological inhibition of STAT6 demonstrated that STAT6 is necessary for IL-4-mediated suppression of pro-inflammatory microglial activity and preservation of endothelial barrier function (**Figure 5D**; **Supplementary Figures 5A**, **8A–D**). These effects may also contribute to anti-angiogenic outcomes, as regulatory microglia and preserved pericytes are known to inhibit neovascularization (46, 47). Together, these findings suggest that IL-4, via STAT6-mediated modulation of microglial activity, may help limit neovascularization in DR by protecting pericytes and reducing inflammatory cytokine production.

Beyond its mechanistic insights, our findings carry important implications for the therapeutic landscape of DR. While current DR treatments, such as anti-VEGF therapy or laser photocoagulation, primarily target advanced stages of the disease (48), these approaches do not address the early pathogenic events like pericyte apoptosis and chronic neuroinflammation that drive vascular damage. The ability of IL-4 to concurrently enhance pericyte survival and attenuate microglial inflammatory signaling suggests it may have disease-modifying potential when administered during earlier stages of DR. Furthermore, our co-culture models underscore the context-dependent nature of the effects of IL-4 and highlight the neurovascular unit, rather than individual cell types, as a more appropriate therapeutic target.

In conclusion, our study demonstrates that IL-4—whose levels are decreased in diabetic retinas—promotes pericyte survival through two complementary mechanisms: directly activating the PI3K/Akt pathway in pericytes and indirectly modulating microglial inflammatory status via STAT6 activation. These dual actions of IL-4 contribute to preventing increased endothelial permeability under diabetic conditions. Collectively, our results identify IL-4 as a critical regulator of retinal vascular integrity in DR and suggest its potential as a therapeutic target to preserve pericytes and maintain endothelial barrier function. Given that vascular leakage and inflammation are central to DR progression, IL-4-based therapies may provide a novel strategy to prevent or delay vision loss by stabilizing the retinal microvasculature and modulating neuroinflammation. Furthermore, owing to its pleiotropic roles in immune modulation and tissue repair, IL-4 may also have broader therapeutic relevance for other neurovascular disorders characterized by endothelial dysfunction and glial activation. Future studies are needed to investigate whether IL-4 delivery, through intravitreal injection, gene therapy, or sustained-release formulations, can restore vascular stability and slow disease progression *in vivo* and to evaluate the translational potential of IL-4 in preclinical models and clinical trials.

## Data Availability

The original contributions presented in the study are included in the article/supplementary material. Further inquiries can be directed to the corresponding author.

## Glossary

DR: Diabetic Retinopathy
IL4: interleukin 4
STZ: streptozotocin
qRT-PCR: quantitative reverse transcription polymerase chain reaction
Con: control
ELISA: enzyme-linked immunosorbent assay
pSTAT6: phosphorylated signal transducer and activator of transcription 6
STAT6: signal transducer and activator of transcription 6
pSTAT5: phosphorylated signal transducer and activator of transcription 5
STAT5: signal transducer and activator of transcription 5
pAkt: phosphorylated protein kinase B
Akt: protein kinase B
pErk1/2: phosphorylated extracellular signal-regulated kinase 1/2
Erk1/2: extracellular signal-regulated kinase 1/2
β-tubulin: beta-tubulin. TNF-α, tumor necrosis factor-alpha
MTT: (3-(4,5-dimethylthiazol-2-yl)-2,5-diphenyltetrazolium bromide) assay
PI: propidium iodide
Bax: Bcl-2-associated X protein
Bcl-2: B-cell lymphoma 2
Bcl-xL: B-cell lymphoma-extra-large
FBS: fetal bovine serum
DMEM: Dulbecco’s Modified Eagle’s medium
ZO-1: Zona Occludens
Arg-1: arginase-1
IGF-1: insulin-like growth factor 1
MAPK: mitogen activated protein kinase.
