# Supplementary material for: Interleukin-4 prevents increased endothelial permeability by inducing pericyte survival and modulating microglial responses in diabetic retinopathy
Source: Front Endocrinol (Lausanne). 2025 Jul 2;16:1609796. doi: 10.3389/fendo.2025.1609796 (PMC12263392; doi:10.3389/fendo.2025.1609796)
Supplement: Supplementary file 2 [file DataSheet2.pdf]

Table 1. Primer sequences used for qPCR analysis.

Gene	Species	Forward Primer (5'–3')	Reverse Primer (5'–3')
Il4	Mouse	ATGGGTCTCAACCCCCAGCTAGT	GCTCTTTAGGCTTTCCAGGAAGTC
Ilb	Mouse	CCCATTAGACAACTGCACTAC	GATTCTTTCCTTTGAGGCCC
Il6	Mouse	CTTCTTGGGACTGATGCTGGT	GGTCTGTTGGGAGTGGTATCC
Il10	Mouse	CGGGAAGACAATAACTG	CATTTCCGATAAGGCTTGG
Il12b	Mouse	ATCGTTTTGCTGGTGTCTCC	CATCTTCTTCAGGCGTGTCA
Il13	Mouse	CCTCTGACCCTTAAGGAGCTT	ATGTTGGTCAGGGAATCCAG
Il18	Mouse	ACAACTTTGGCCGACTTCAC	GGGTTCACTGGCACTTTGAT
Actb	Mouse	CCAGGCATTGCTGACAGGAT	AGCCACCGATCCACACAGAG
ILB	Human	ACGCTCCGGGACTCACAGCA	TGAGGCCCAAGGCCACAGGT
IL6	Human	TGACAAACAAATTCGGTACATCCT	AGTGCCTCTTTGCTGCTTTCAC
IL23A	Human	GTGGGACACATGGATCTAAGAGAAG	TTTGCAAGCAGAACTGACTGTTG
TNFA	Human	CACAGTGAAGTGCTGGCAAC	AGGAAGGCCTAAGGTCCACT
ARG1	Human	TGGACAGACTAGGAATTGGCA	CCAGTCCGTCAACATCAAAACT
IL10	Human	GACTTTAAGGGTTACCTGGGTTG	TCACATGCGCCTTGATGTCTG
IGF1	Human	TGTGGAGACAGGGGCTTTTA	CCTGCACTCCCTCTACTTGC
ACTB	Human	GGGAAATCGTGCGTGACATT	AGTTTCGTGGATGCCACAGG
