# Supplementary material for: Interleukin-4 prevents increased endothelial permeability by inducing pericyte survival and modulating microglial responses in diabetic retinopathy
Source: Front Endocrinol (Lausanne). 2025 Jul 2;16:1609796. doi: 10.3389/fendo.2025.1609796 (PMC12263392; doi:10.3389/fendo.2025.1609796)
Supplement: Supplementary file 4 [file DataSheet4.pdf]

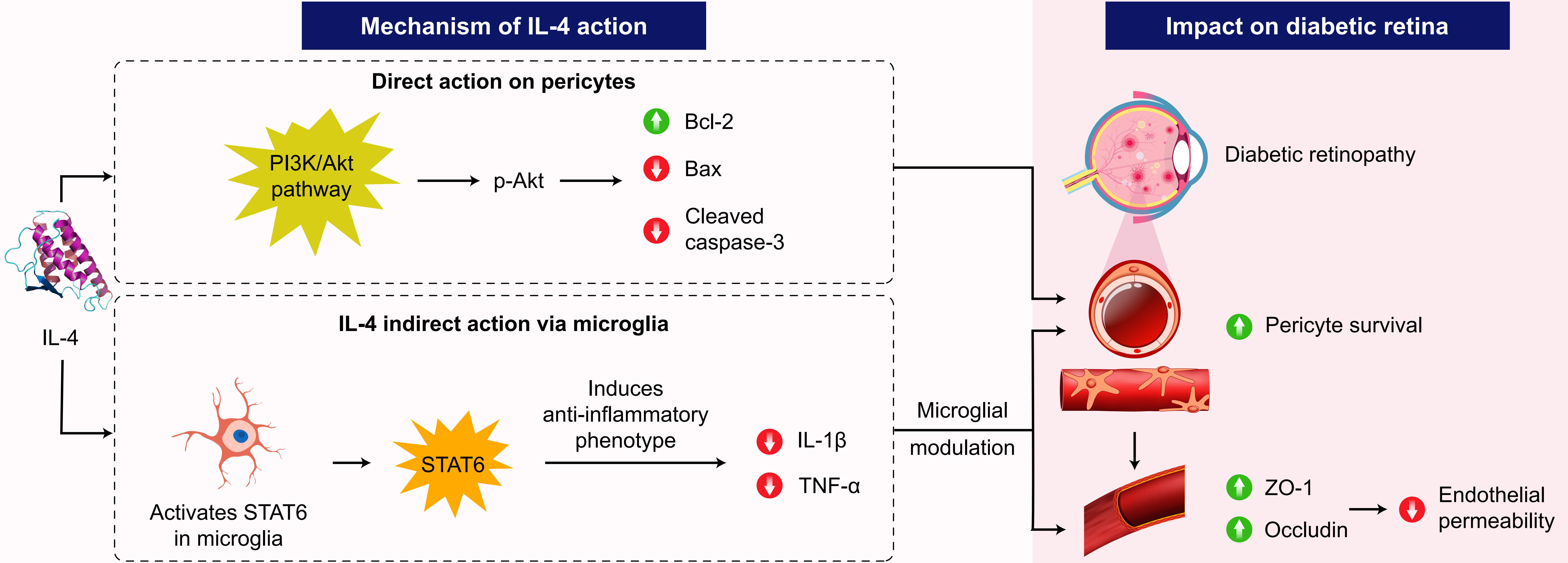

PI3K/Akt: phosphoinositide 3-kinase/protein kinase; IL: interleukin; STAT6: signal transducer and activator of transcription 6; Bcl-2: B-cell lymphoma; Bax: Bcl-2-associated X protein; ZO-1: zonula occludens-1; TNF-α: tumor necrosis factor-alpha
