# Supplementary figures and images for: Interleukin-4 prevents increased endothelial permeability by inducing pericyte survival and modulating microglial responses in diabetic retinopathy
Source: Front Endocrinol (Lausanne). 2025 Jul 2;16:1609796. doi: 10.3389/fendo.2025.1609796 (PMC12263392; doi:10.3389/fendo.2025.1609796)

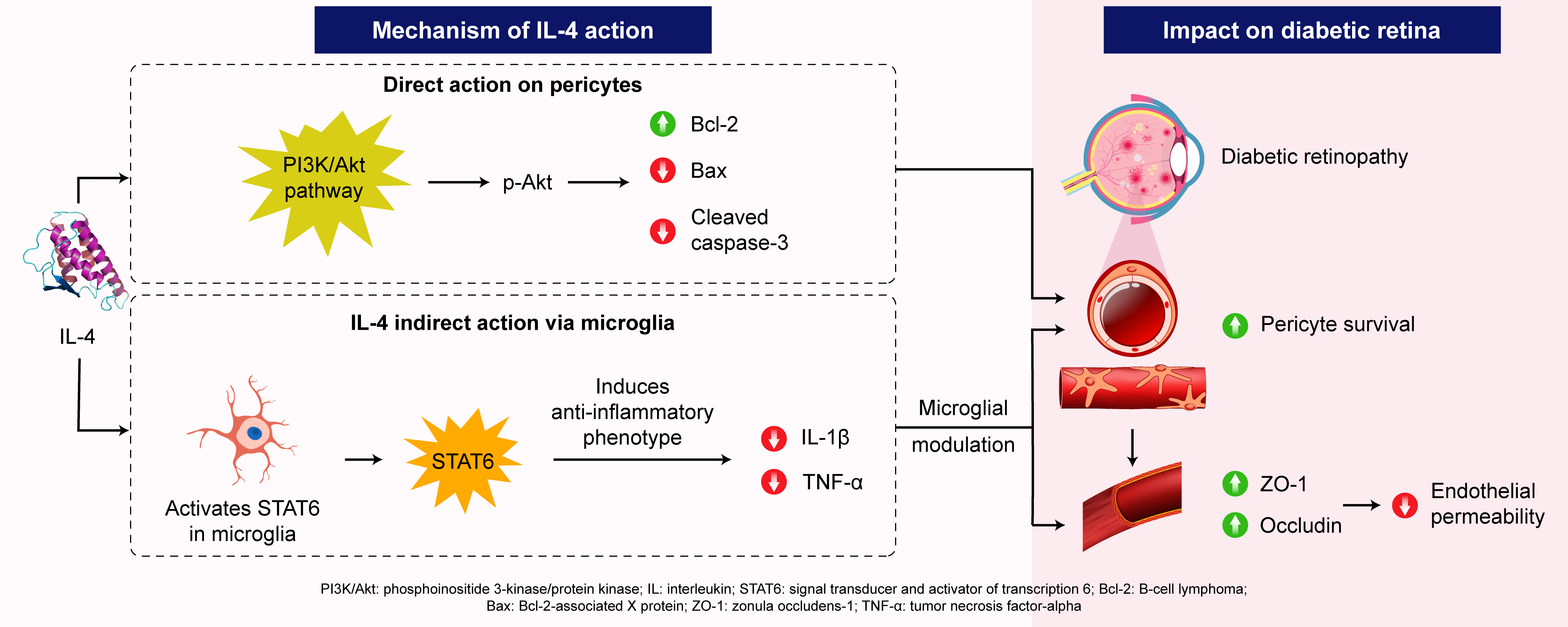

Supplement: Supplementary file 5 [file Image1.tif]
