# Supplementary material for: Interleukin-4 prevents increased endothelial permeability by inducing pericyte survival and modulating microglial responses in diabetic retinopathy
Source: Front Endocrinol (Lausanne). 2025 Jul 2;16:1609796. doi: 10.3389/fendo.2025.1609796 (PMC12263392; doi:10.3389/fendo.2025.1609796)
Supplement: Supplementary file 6 [file Table1.docx]

Table 1. Primer sequences used for qPCR analysis.

| Gene | Species | Forward Primer (5'–3') | Reverse Primer (5'–3') |
| --- | --- | --- | --- |
| Il4 | Mouse | ATGGGTCTCAACCCCCAGCTAGT | GCTCTTTAGGCTTTCCAGGAAGTC |
| Ilb | Mouse | CCCATTAGACAACTGCACTAC | GATTCTTTCCTTTGAGGCCC |
| Il6 | Mouse | CTTCTTGGGACTGATGCTGGT | GGTCTGTTGGGAGTGGTATCC |
| Il10 | Mouse | CGGGAAGACAATAACTG | CATTTCCGATAAGGCTTGG |
| Il12b | Mouse | ATCGTTTTGCTGGTGTCTCC | CATCTTCTTCAGGCGTGTCA |
| Il13 | Mouse | CCTCTGACCCTTAAGGAGCTT | ATGTTGGTCAGGGAATCCAG |
| Il18 | Mouse | ACAACTTTGGCCGACTTCAC | GGGTTCACTGGCACTTTGAT |
| Actb | Mouse | CCAGGCATTGCTGACAGGAT | AGCCACCGATCCACACAGAG |
| ILB | Human | ACGCTCCGGGACTCACAGCA | TGAGGCCCAAGGCCACAGGT |
| IL6 | Human | TGACAAACAAATTCGGTACATCCT | AGTGCCTCTTTGCTGCTTTCAC |
| IL23A | Human | GTGGGACACATGGATCTAAGAGAAG | TTTGCAAGCAGAACTGACTGTTG |
| TNFA | Human | CACAGTGAAGTGCTGGCAAC | AGGAAGGCCTAAGGTCCACT |
| ARG1 | Human | TGGACAGACTAGGAATTGGCA | CCAGTCCGTCAACATCAAAACT |
| IL10 | Human | GACTTTAAGGGTTACCTGGGTTG | TCACATGCGCCTTGATGTCTG |
| IGF1 | Human | TGTGGAGACAGGGGCTTTTA | CCTGCACTCCCTCTACTTGC |
| ACTB | Human | GGGAAATCGTGCGTGACATT | AGTTTCGTGGATGCCACAGG |
