# Supplementary material for: Interleukin-4 prevents increased endothelial permeability by inducing pericyte survival and modulating microglial responses in diabetic retinopathy
Source: Front Endocrinol (Lausanne). 2025 Jul 2;16:1609796. doi: 10.3389/fendo.2025.1609796 (PMC12263392; doi:10.3389/fendo.2025.1609796)
Supplement: Supplementary file 7 [file Table2.docx]

Supplementary Material

# Supplementary Figures


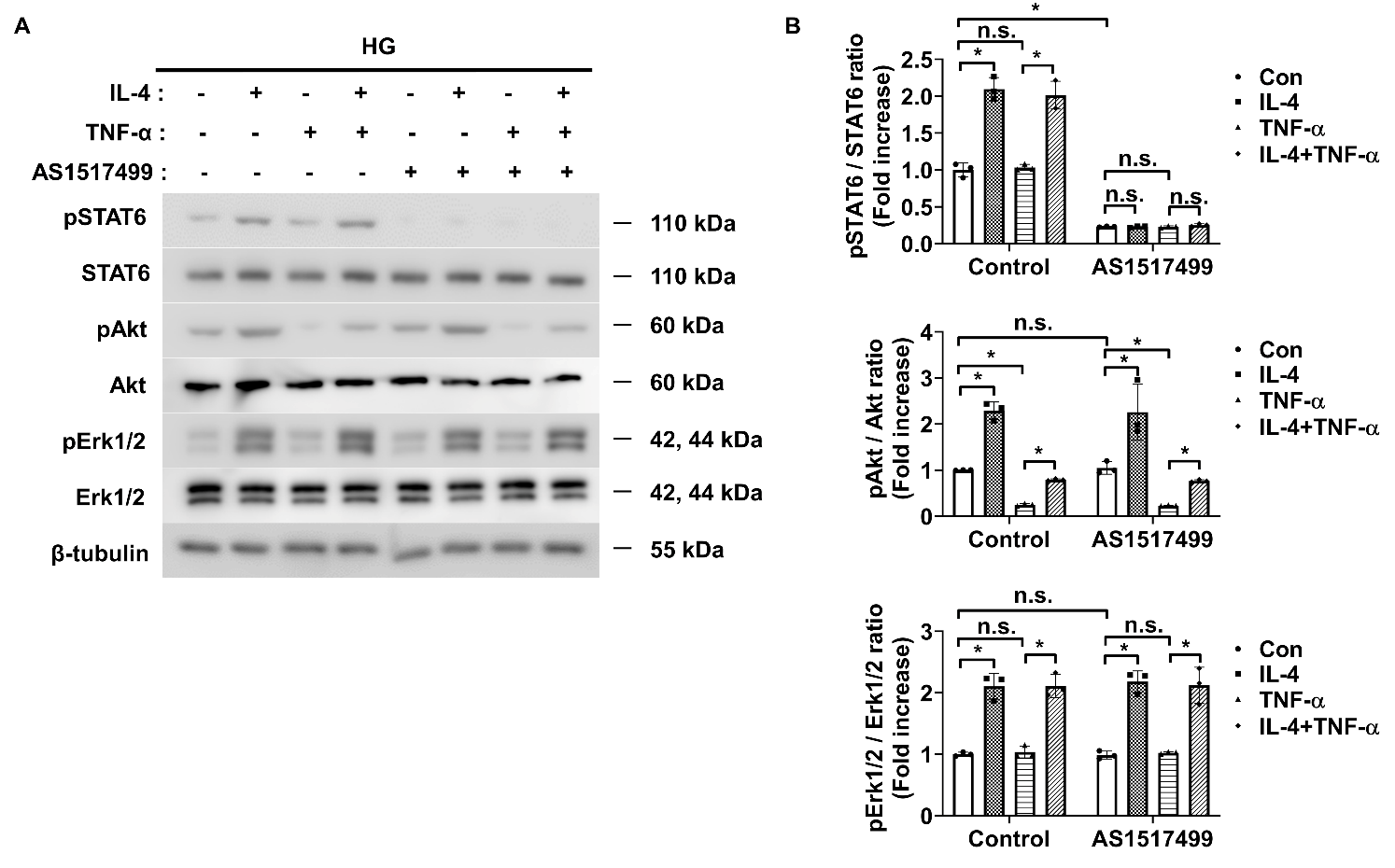


**Supplementary Figure 1.** AS1517499 completely block STAT6 phosphorylation induced by IL-4. (A) Pericytes were preincubated with the AS1517499 (1 μM) for 1 h and treated with or without IL-4 (50 ng/mL) and TNF-α (100 ng/mL) for 30 min. Western blot analysis for phospho-STAT6 (pSTAT6), STAT6, phospho-Akt (pAkt), Akt, phospho-Erk1/2 (pErk1/2), and Erk1/2 were performed on lysates obtained from pericytes under high glucose (HG; 25 mmol/L glucose) conditions. β-tubulin was used as a loading control. (B) Quantitative densitometric analysis in (A) to calculate the ratio of each protein to β-tubulin (*n* = 3). Statistical analysis was performed using two-way ANOVA followed by Tukey’s post hoc test. **P* < 0.05.

Abbreviations: AS1517499, a selective STAT6 inhibitor; IL-4, interleukin-4; TNF-α, tumor necrosis factor-alpha; pSTAT6, phosphorylated signal transducer and activator of transcription 6; STAT6, signal transducer and activator of transcription 6; pAkt, phosphorylated protein kinase B; Akt, protein kinase B; pErk1/2, phosphorylated extracellular signal-regulated kinase 1/2; Erk1/2, extracellular signal-regulated kinase 1/2; β-tubulin, beta-tubulin; HG, high glucose.

**
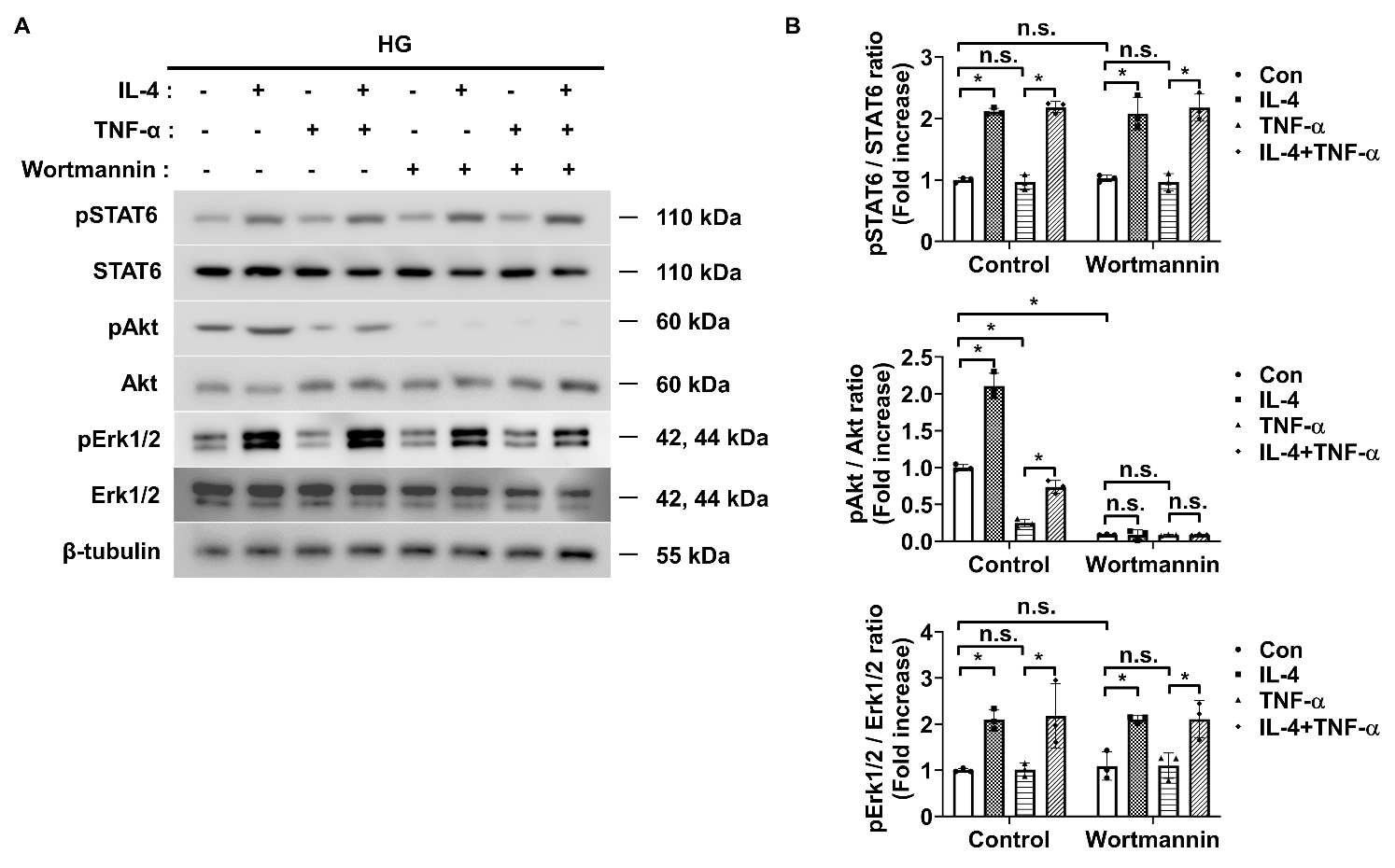
**

**Supplementary Figure 2.** Wortmannin completely blocks Akt phosphorylation induced by IL-4. (A**)** Pericytes were preincubated with the Wortmannin (1 μM) for 1 h and treated with or without IL-4 (50 ng/mL) and TNF-α (100 ng/mL) for 30 min. Western blot analysis for phospho-STAT6 (pSTAT6), STAT6, phospho-Akt (pAkt), Akt, phospho-Erk1/2 (pErk1/2), and Erk1/2 were performed on lysates obtained from pericytes under high glucose (HG; 25 mmol/L glucose) conditions. β-tubulin was used as a loading control. (B) Quantitative densitometric analysis in (A) to calculate the ratio of each protein to β-tubulin (*n* = 3). Statistical analysis was performed using two-way ANOVA followed by Tukey’s post hoc test. **P* < 0.05.

**
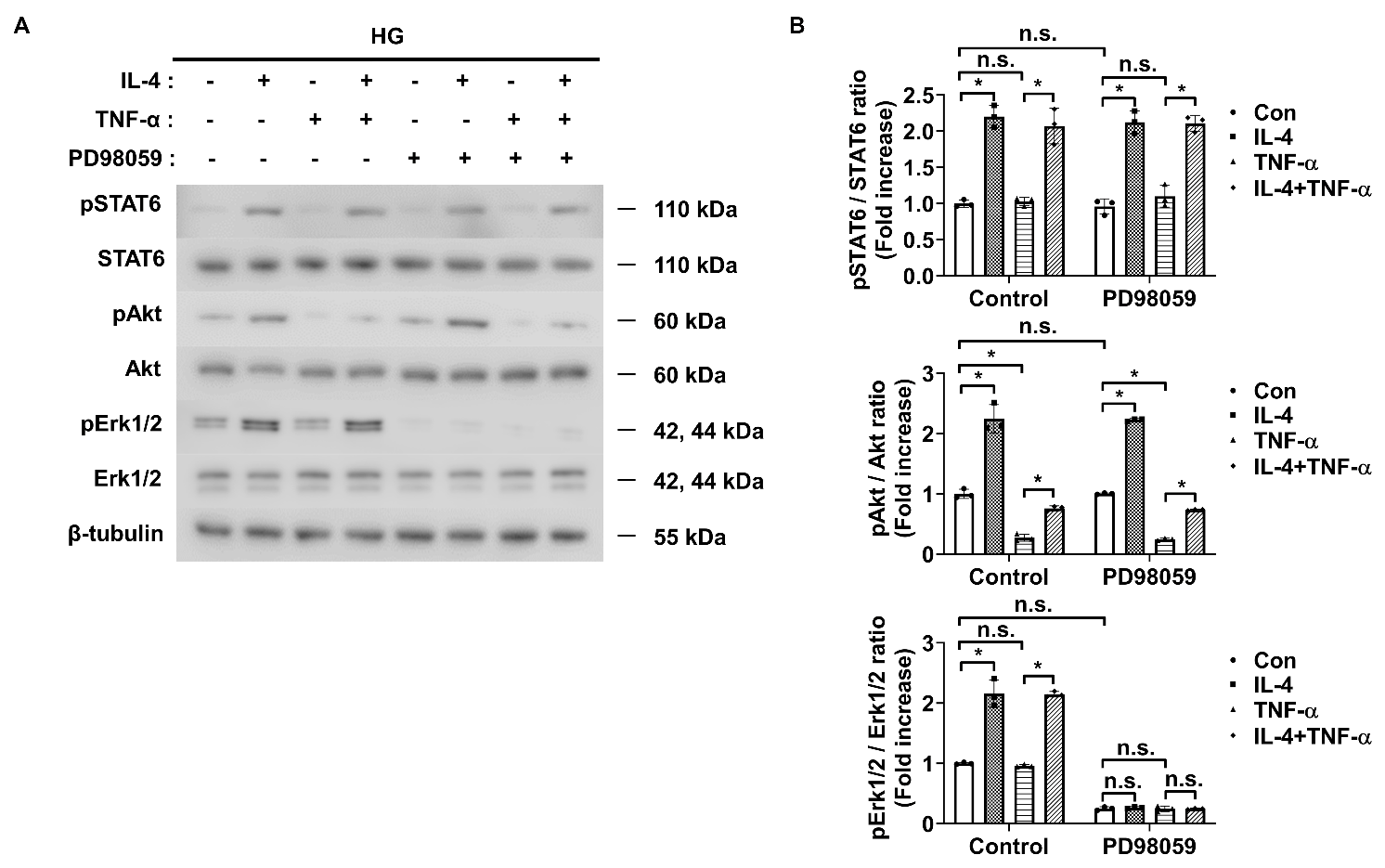
**

**Supplementary Figure 3.** PD98059 completely blocks Erk1/2 phosphorylation induced by IL-4. (A) Pericytes were preincubated with the PD98059 (25 μM) for 1 h and treated with or without IL-4 (50 ng/mL) and TNF-α (100 ng/mL) for 30 min. Western blot analysis for phospho-STAT6 (pSTAT6), STAT6, phospho-Akt (pAkt), Akt, phospho-Erk1/2 (pErk1/2), and Erk1/2 were performed on lysates obtained from pericytes under high glucose (HG; 25 mmol/L glucose) conditions. β-tubulin was used as a loading control. (B) Quantitative densitometric analysis in (A) to calculate the ratio of each protein to β-tubulin (*n* = 3). Statistical analysis was performed using two-way ANOVA followed by Tukey’s post hoc test. **P* < 0.05.


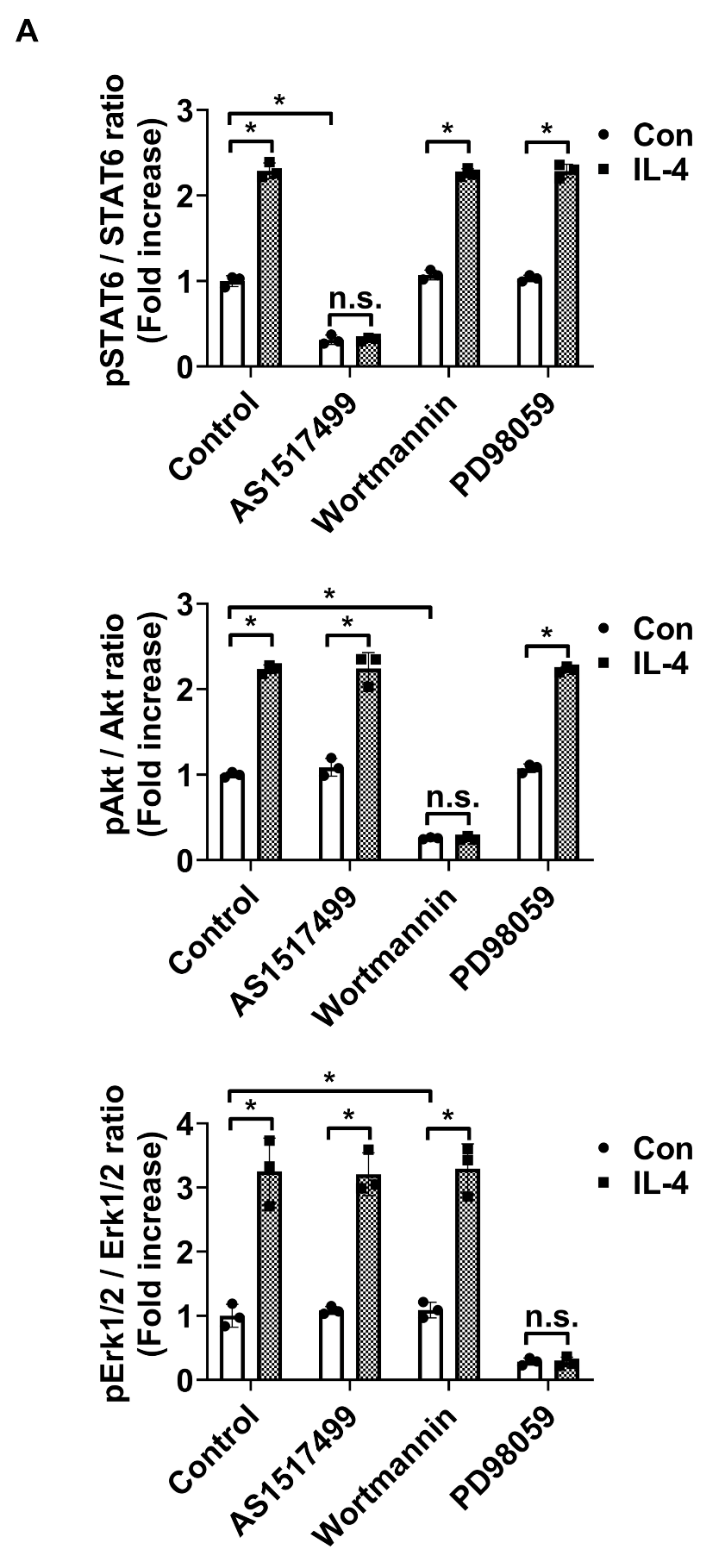


**Supplementary Figure 4.** AS1517499, Wortmannin, and PD98059 completely block STAT6, Akt, and Erk1/2 phosphorylation induced by IL-4 in HMO6 cells, respectively. (A) Quantitative densitometric analysis in Figure 5C to calculate the ratio of each protein to β-tubulin (*n* = 3). Statistical analysis was performed using two-way ANOVA followed by Tukey’s post hoc test. **P* < 0.05.

**
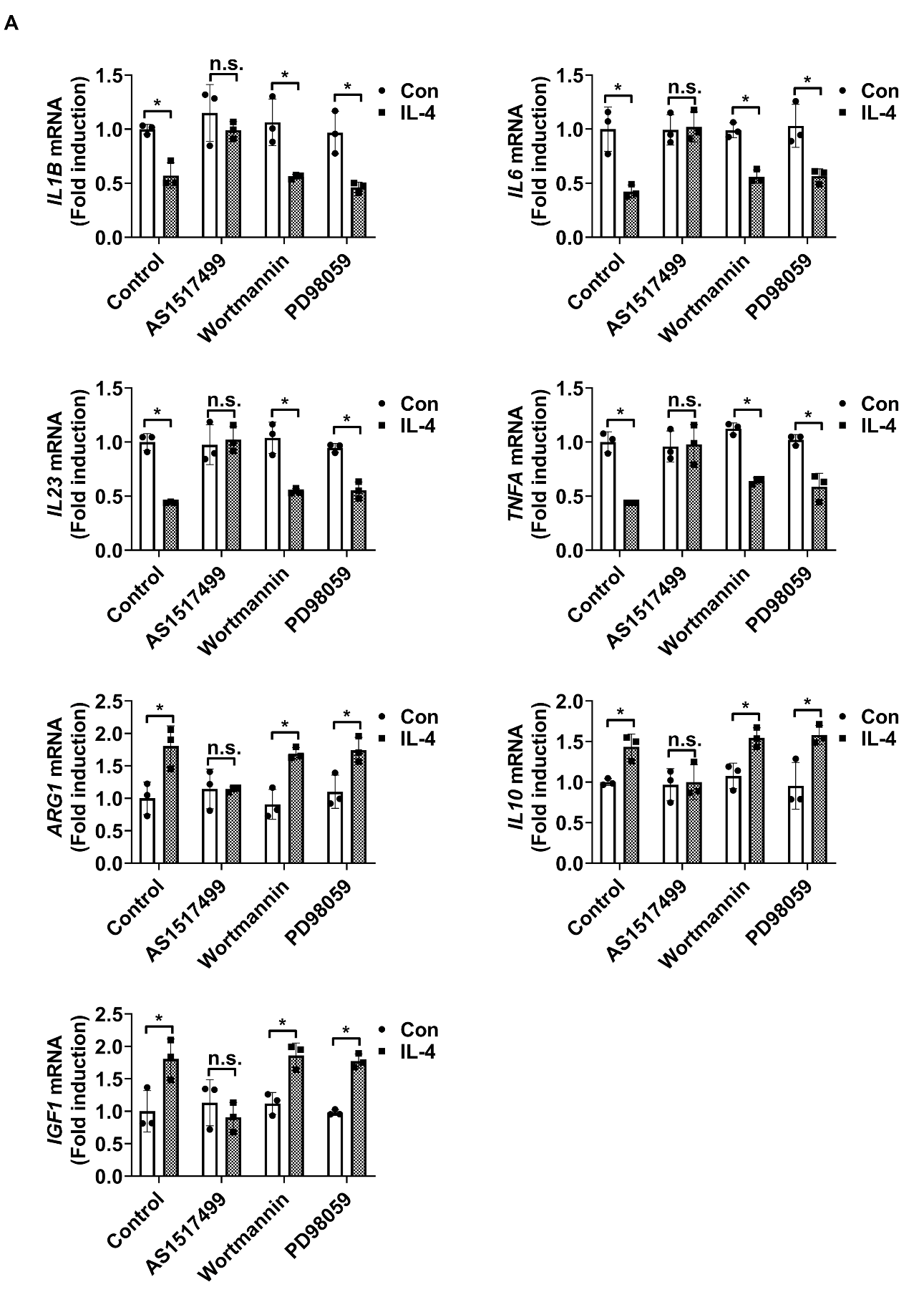
**

**Supplementary Figure 5.** IL-4 modulates microglial responses via STAT6 signaling. (A) HMO6 cells were preincubated with the AS1517499, Wortmannin, or PD98059 for 1 h and treated with or without IL-4 for 48 h under high glucose conditions. The mRNA levels of *IL1B, IL6, IL23, TNFA*, *Arg1*, *IL10*, and *IGF1* were determined by qRT-PCR. The bar graph represents mean ± SD (*n* = 3). Statistical analysis was performed using two-way ANOVA followed by Tukey’s post hoc test. n.s, not significant; **P* < 0.05.

Abbreviations: HMO6, human microvascular endothelial cells; Arg-1, arginase-1; IGF-1, insulin-like growth factor 1; qRT-PCR, quantitative reverse transcription polymerase chain reaction.


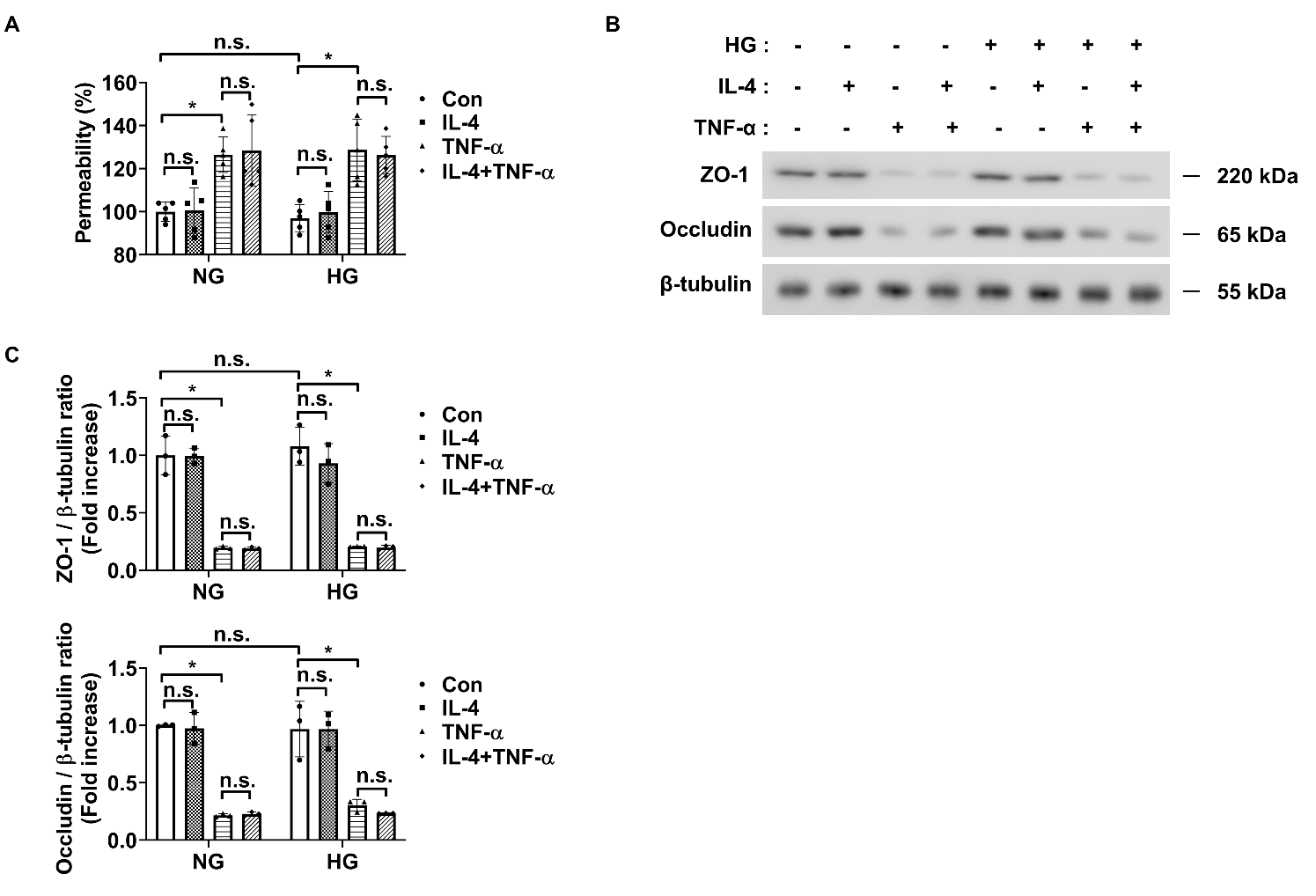


**Supplementary Figure 6.** IL-4 does not directly affect the permeability of endothelial cells. (A, B) HRMECs were exclusively cultured on the top side of the Transwell inserts. HRMECs were exposed to the conditions of normal glucose (NG; 5 mmol/L glucose) or high glucose (HG; 25 mmol/L glucose) conditions with or without IL-4 (50 ng/ml) and TNF-α (100 ng/ml) for 48 h. (A) The permeability was measured with Evans blue dye (n = 5). Statistical analysis was performed using two-way ANOVA followed by Tukey’s post hoc test. n.s, not significant; **P* < 0.05. (B) Western blot analysis for ZO-1 and occludin performed in lysates obtained from (A). β-tubulin was used as a loading control. (C) Quantitative densitometric analysis in (B) to calculate the ratio of each protein to β-tubulin (n = 3). Statistical analysis was performed using two-way ANOVA followed by Tukey’s post hoc test. n.s, not significant; **P* < 0.05.

Abbreviations: HRMECs, human retinal microvascular endothelial cells.


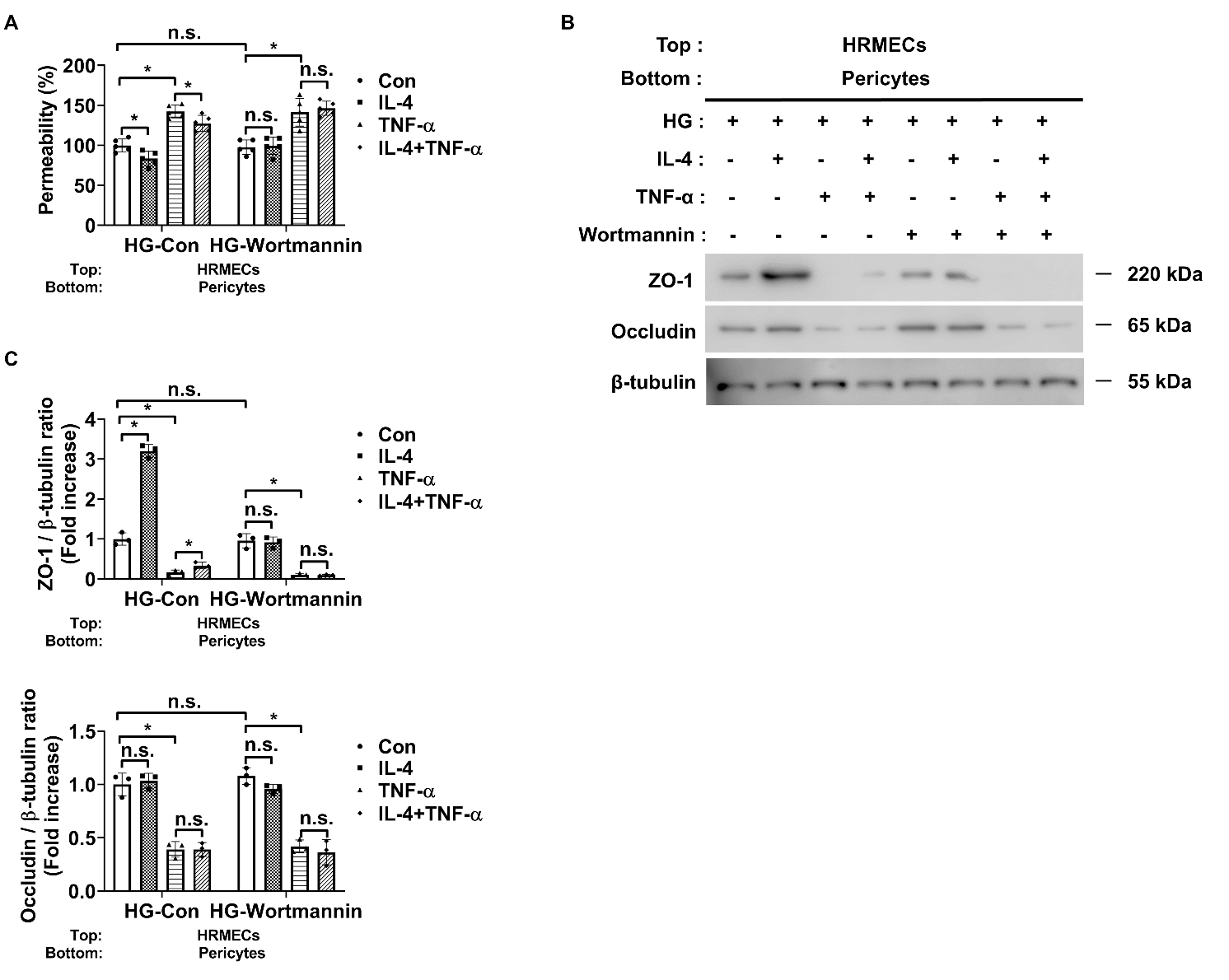


**Supplementary Figure 7.** IL-4 prevents the increase in endothelial permeability through pericyte survival during DR. (A-B) Pericytes and HRMECs were co-cultured on the indicated sides of the Transwell inserts. The cells were preincubated with Wortmannin (1 μM) for 1 h and then exposed to high glucose (HG; 25 mmol/L glucose) conditions with or without IL-4 (50 ng/ml) and TNF-α (100 ng/ml) for 48 h. (A) The permeability was measured with Evans blue dye (n = 5). Statistical analysis was performed using two-way ANOVA followed by Tukey’s post hoc test. n.s, not significant; **P* < 0.05. (B) Western blot analysis for ZO-1 and occludin performed in lysates obtained from HRMECs in (A). β-tubulin was used as a loading control. (C) Quantitative densitometric analysis in (B) to calculate the ratio of each protein to β-tubulin (n = 3). Statistical analysis was performed using two-way ANOVA followed by Tukey’s post hoc test. n.s, not significant; **P* < 0.05.


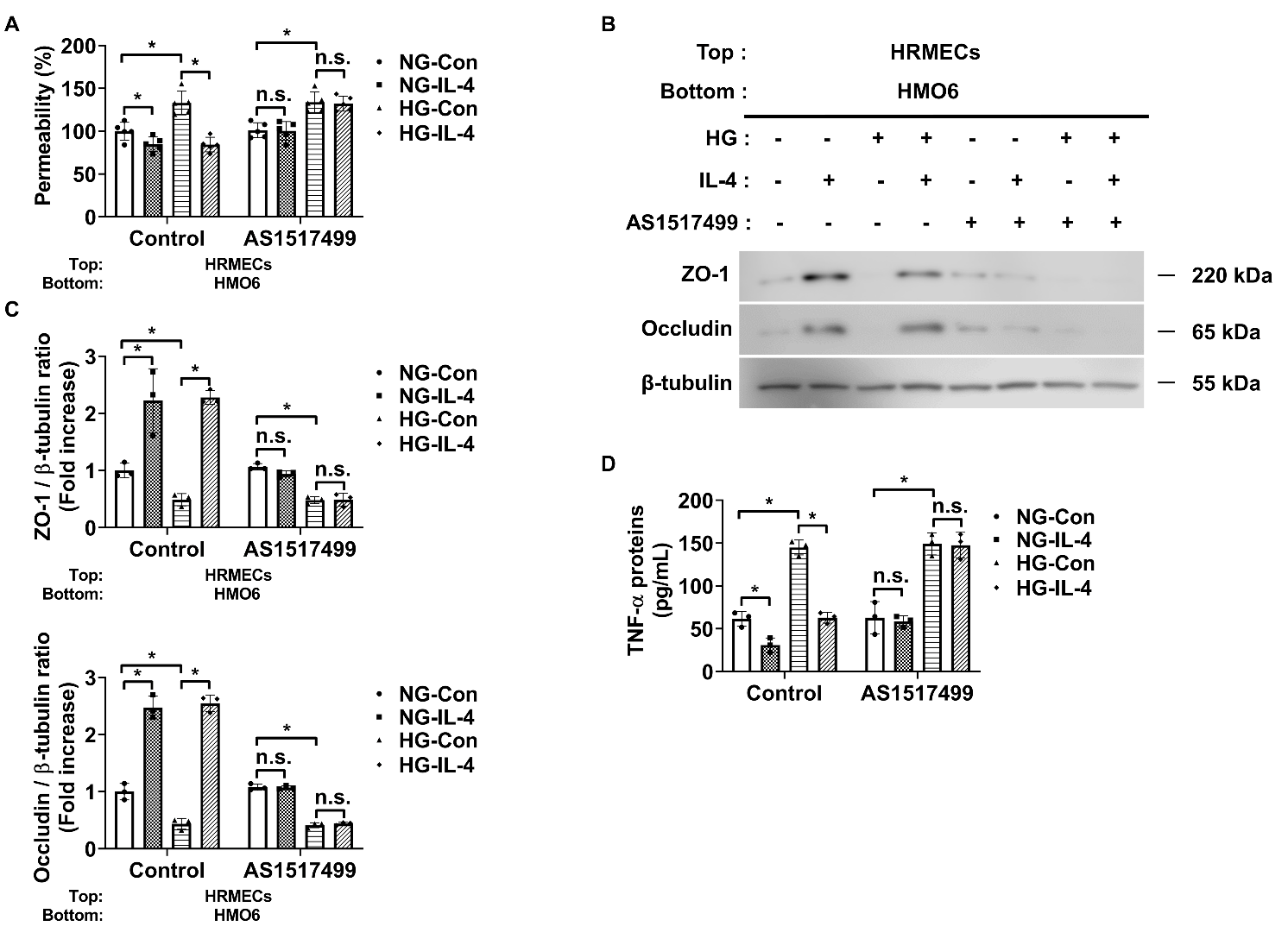


**Supplementary Figure 8.** IL-4 reduces endothelial permeability during DR by modulating microglial responses. (A-B) HMO6 cells and HRMECs were co-cultured on the indicated sides of the Transwell inserts. The cells were preincubated with AS1517499 (1 μM) for 1 h and then exposed to normal glucose (NG; 5 mmol/L glucose) or high glucose (HG; 25 mmol/L glucose) conditions with or without IL-4 (50 ng/ml) for 48 h. (A) The permeability was measured with Evans blue dye (n = 5). Statistical analysis was performed using two-way ANOVA followed by Tukey’s post hoc test. n.s, not significant; **P* < 0.05. (B) Western blot analysis for ZO-1 and occludin performed in lysates obtained from HRMECs in (A). β-tubulin was used as a loading control. (C) Quantitative densitometric analysis in (B) was used to calculate the ratio of each protein to β-tubulin (n = 3). Statistical analysis was performed using two-way ANOVA followed by Tukey’s post hoc test. n.s, not significant; **P* < 0.05. (D) The protein levels of TNF-α in conditioned media obtained after the experiment were determined by ELISA. The bar graph represents mean ± SD (*n* = 3). Statistical analysis was performed using two-way ANOVA followed by Tukey’s post hoc test. n.s, not significant; **P* < 0.05.

ELISA, enzyme-linked immunosorbent assay; ZO-1, zonula occludens-1.
